# Kinome analysis of *Madurella mycetomatis* identified kinases in the cell wall integrity pathway as novel potential therapeutic drug targets in eumycetoma caused by *Madurella mycetomatis*

**DOI:** 10.1371/journal.pntd.0013482

**Published:** 2025-09-04

**Authors:** Mickey Konings, Nikolaos Strepis, Ri-Ichiroh Manabe, Akira Hasegawa, Somdatta Chaudhari, Saskia du Pré, Marij Schippers, Michihira Tagami, Jingyi Ma, Yasushi Okazaki, Matthew Todd, Bernhard Biersack, Vijay Masand, Annelies Verbon, Takeya Kasukawa, Imad Abugessaisa, Wendy W. J. van de Sande

**Affiliations:** 1 Department of Medical Microbiology and Infectious Diseases Rotterdam, Erasmus Medical Center, University Medical Centre, Rotterdam, The Netherlands; 2 Laboratory for Comprehensive Genomic Analysis, RIKEN Center for Integrative Medical Sciences, Yokohama, Kanagawa, Japan; 3 Laboratory for Large-Scale Biomedical Data Technology, RIKEN Center for Integrative Medical Sciences, Yokohama, Kanagawa, Japan; 4 Department of Pharmaceutical Chemistry, Progressive Education society’s Modern College of Pharmacy, Pune, Maharashtra, India; 5 School of Pharmacy and Structural Genomics Consortium, University College London, London, United Kingdom; 6 Organic Chemistry Laboratory, University Bayreuth, Bayreuth, Germany; 7 Department of Chemistry, Vidya Bharati Mahavidyalaya, Amravati, Maharashtra, India; 8 Department of Internal Medicine, UMC Utrecht, Utrecht, The Netherlands; 9 Premium Research Institute for Human Metaverse Medicine (WPI-PRIMe), The University of Osaka, Suita, Osaka, Japan; 10 Graduate School of Medicine and Faculty of Medicine, The University of Osaka, Suita, Osaka, Japan; Erasmus Medical Center, Rotterdam University, NETHERLANDS, KINGDOM OF THE

## Abstract

Eumycetoma is a neglected tropical subcutaneous disease most commonly caused by the fungus *Madurella mycetomatis.* Currently, eumycetoma is treated by a combination of antifungal therapy and surgery, with limited success rates. To identify novel drug targets we used an *in silico* approach to determine the kinases present in *M. mycetomatis* genome and rank them as potential drug targets. In total 132 predicted kinases were identified in *M. mycetomatis*, of which 21 were predicted to be essential for fungal viability and 4 of these had no human orthologues. Two were linked to the Cell Wall Integrity (CWI) signalling pathway and were expressed in a *Galleria mellonella* infection model. Several kinase inhibitors were identified after *in silico* modelling, however only 8 were able to inhibit growth. Five had predicted binding affinity with components of the CWI. Altogether, the CWI shows potential as a drug target for further evaluation.

## Author summary

Eumycetoma is a neglected tropical infection of the skin. It is characterised by large tumorous lesions and very difficult to treat. Even after treatment for more than a year and surgical removal of the lesion, many patients are not cured. There is therefore an urgent need to find better drugs for eumycetoma. In the past new drugs were usually identified by screening large drug libraries to determine if compounds were active against the most common causative agent of eumycetoma, the fungus *Madurella mycetomatis.* However, nowadays we have the genome of *M. mycetomatis* available from which we can identify drug targets *in silico.* By then performing transcriptomic analysis, we can further determine if these potential drug targets are expressed during infection. In this study we focused on kinases, enzymes that catalyse protein phosphorylation processes that are involved in a wide variety of important signalling pathways, regulating cellular processes such as proliferation, differentiation, and stress response, that are vital for cell survival, adaptation and virulence. By comparative genomics we identified 132 predicted kinases in *M. mycetomatis*, of which 21 were predicted to be essential for fungal viability and 4 of these had no human orthologues. Two of these four kinases were linked to the Cell Wall Integrity (CWI) signalling pathway and were expressed in a *Galleria mellonella* infection model. Several kinase inhibitors were identified after *in silico* modelling, however only 8 were able to inhibit growth. Five had predicted binding affinity with components of the CWI. Altogether, the CWI shows potential as a drug target for further evaluation.

## Introduction

Mycetoma is a chronic infection of the subcutaneous tissue, characterized by large tumorous lesions especially on the foot. In the past, this disease was therefore also referred to as Madura foot [1]. The disease usually develops after implantation of the causative agent in the subcutaneous tissue via a minor trauma after which a nodule will form [2]. This nodule will increase in size, leading to a tumorous mass. This mass hinders 55% of patients in their daily activities and 17% of patients consequently will lose their employment. Eventually 9% of patients will become completely dependent on their family [3]. The disease is most common in rural areas in tropical and sub-tropical regions in Africa, Latin America and Asia [2,4], with an estimated prevalence of 55 cases per 10,000 inhabitants for Sudan [5]. In 2016, it became recognized as a neglected tropical disease by the World Health Organization (WHO) [6,7].

Mycetoma can be either of bacterial or fungal origin, referred to as actinomycetoma and eumycetoma, respectively. The causative agents are found in the infected tissue in the form of mycetoma grains [8]. In 2023, the eumycetoma causative agents were ranked as high priority fungal pathogens by the WHO [9]. More than 90 different micro-organisms have been indicated as causative agents, but the fungus *Madurella mycetomatis* is the most common as it was reported in 10,556 cases, representing over 85% of all eumycetoma cases [2,10]. Treatment depends on the causative agent. Actinomycetoma can be treated with antimicrobial agents only, with cure rates as high as 90% [11]. Eumycetoma in contrast, cannot be treated by medication alone. The standard treatment consists of six months antifungal treatment with itraconazole, followed by surgical removal of the lesion and then at least another six months of itraconazole to prevent recurrence [12]. In a clinical trial setting, this resulted in a cure rate of 80% [13]. In normal clinical practice, corrected cure rates of 67.6% were reported, with 6.1% of patients needing to undergo amputation [10,13]. Notably, these cure rates are in combination with surgery, and in the first six months of treatment no reduction of the lesion size or a decrease in the fungal infection marker levels of beta-glucan is observed [13,14]. Therefore, exploring alternatives to the currently available antifungal agents is imperative to enhance therapeutic outcomes for *M. mycetomatis* infections, ideally eliminating the need for surgical intervention.

Drug discovery efforts typically start with the identification of suitable drug targets or drug candidates, via phenotypic high throughput screenings, in order to identify lead compounds [15]. While this methodology allows for screening large numbers of compounds for antimicrobial activity, it uses an untargeted approach that requires rigorous testing and many resources. In recent years, the increased availability of omics data has enabled the implementation of alternative strategies in the search for new antifungal agents. One such strategy involves the use of bioinformatical analysis of genomic data to identify novel drug targets [16]. A recent example of this strategy is the exploration of the fungal kinome. Kinases are enzymes that catalyse protein phosphorylation processes that are involved in a wide variety of important signalling pathways, regulating cellular processes such as proliferation, differentiation, and stress response, that are vital for cell survival, adaptation and virulence [17]. Therefore, in an effort to interfere with these pivotal processes in eukaryotic systems, fungal genomes have been mined to identify encoded kinases and assess the drugability of these targets [18]. An example of this is the dual-specificity tyrosine-regulated kinase (DYRK) Pom1/YakA, for which inhibition blocked filamentation and biofilm formation in *Candida albicans*, impacted septal plugging in *Aspergillus fumigatus*, and deletion resulted in smaller lesion sizes in infected mice in an *Aspergillus nidulans* lung model [19–21]. Recent screening of the Medicines for Malaria Venture (MMV) open access drug libraries, identified MMV1804559, a predicted dual-specificity tyrosine-regulated kinase 1 (DYRK1) inhibitor, as a novel lead compound for the Open Source Mycetoma (MycetOS) drug discovery program. MMV1804559 significantly reduced the number of grains in a *M. mycetomatis* grain model in *Galleria mellonella* larvae [22], suggesting that targeting *M. mycetomatis* kinases might potentially be a viable target for further drug discovery efforts [23].

**Fig 1:**
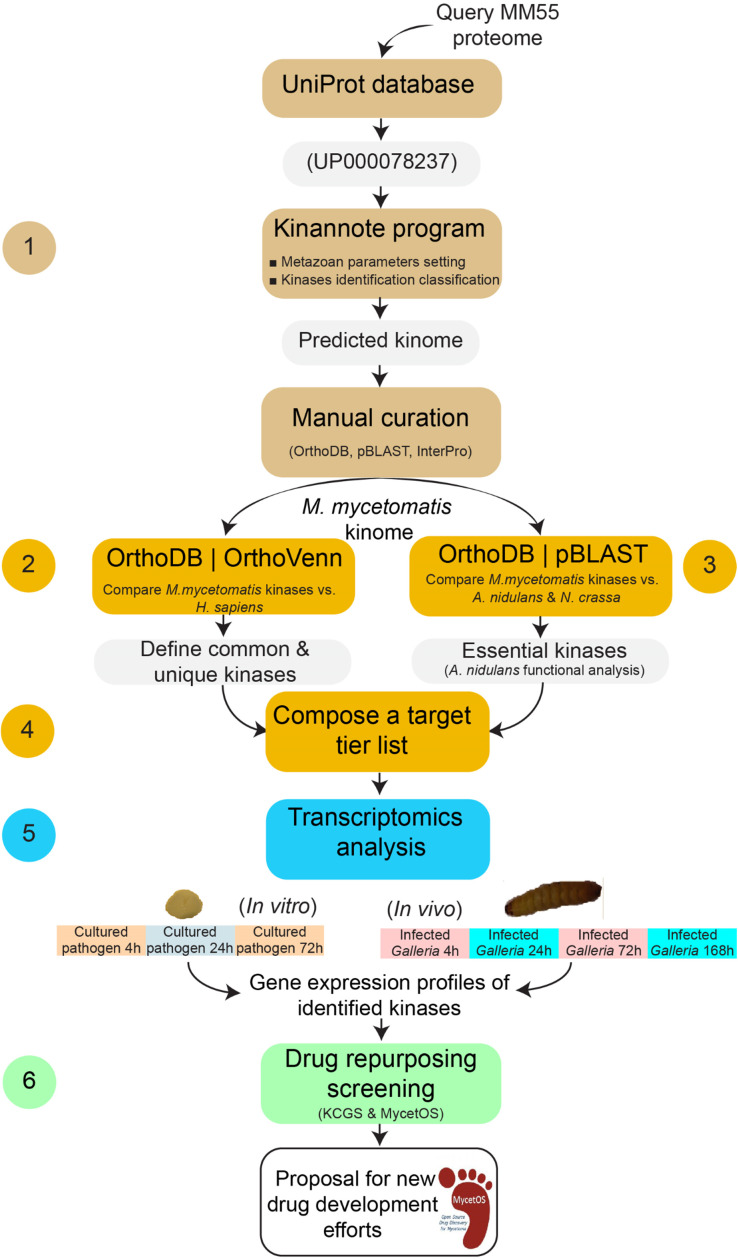
Identification of *M. mycetomatis* kinases as drug targets. In this study, we started by identifying the *M. mycetomatis* kinome by the Kinannote program and manual curation (step 1). To determine which *M. mycetomatis* kinases were absent in the human kinome the list generated in step 1 was compared to the human kinome using OrthoDB and OrthoVenn3 (step 2). In step 3, we compared the *M. mycetomatis* kinome with the kinomes of *A. nidulans* and *N. crassa.* Since for *A. nidulans* it is known which kinases are essential, we used this data to predict which of the *M. mycetomatis* kinases would likely be essential for fungal viability. In step 4 we combined the data from steps 1, 2 and 3 to compose a target tier list based on the orthologue analysis. In step 5, we assessed if the kinases in our target tier list were expressed *in vitro* and *in vivo* in a *M. mycetomatis* grain model in *Galleria mellonella* larvae. In step 6 we screened the kinase inhibitors present in the KCGS library and those donated to the MycetOS project to assess if any of these inhibitors would be able to inhibit *M. mycetomatis* growth. Combined this data would then lead to the identification of new drug target for future drug development efforts in MycetOS.

Therefore, in this study, we aimed to identify *M. mycetomatis* specific kinases which could be potential drug targets to be further investigated in the MycetOS project. For this we screened the genome of *M. mycetomatis* to identify the relevant kinome and compared this first to the human kinome to establish which kinases were absent in the latter. We then compared these *M. mycetomatis* specific kinases to the kinome of *A. nidulans* to determine which of the identified kinases were shared and predicted as essential for fungal viability. To determine which of the kinases were expressed during a *M. mycetomatis* infection in a *G. mellonella* grain model, the gene expression of these kinases was evaluated both *in vitro* and *in vivo* in a *M. mycetomatis* grain model in *G. mellonella* larvae. Finally, the Kinase Chemogenomic Set (KCGS) kinase inhibitor drug library, as well as a set of kinase inhibitors provided by the MycetOS project, were screened *in silico* and *in vitro* to evaluate their ability to bind to the selected kinases and inhibit fungal growth. This with the final goal to identify potential lead compounds for further optimization within the MycetOS program (Fig 1) [24,25].

## Methods

### Defining the *Madurella mycetomatis* kinome

The kinome of *M. mycetomatis* was defined following a previously described bioinformatics pipeline [26]. First, the proteome FASTA sequences (UP000078237), based on the *M. mycetomatis* MM55 genome annotation (PRJNA267680), were extracted from the UniProt database and uploaded to the Kinannote program [27–29]. Here, the metazoan parameter was selected, and the proteome was analysed for the identification of kinases, and further classified in group, family and subfamily. Uncharacterized proteins were identified by InterPro functional domain prediction, protein-BLAST comparison and orthologue identification using the OrthoDB v11 database based on closely related species *A. nidulans* and *Neurospora crassa* [30]. Protein kinase candidates which scored below the threshold as defined by the Kinannote program were labelled as ‘subthreshold’, and were rejected as protein kinases [28].

### Common kinases with the human kinome

To identify kinases of *M. mycetomatis* which were not present in human, the kinome sequences of *M. mycetomatis* and *Homo sapiens* (http://kinase.com/human/kinome/) were evaluated using a comparative genomic approach. First, OrthoVenn3 was used according to the tools documentation with default settings, to identify orthologue clusters and evaluate phylogenetic relationships [31]. Additionally, predicted *M. mycetomatis* kinases were manually compared to human kinases using OrthoDB v11 database [32].

### Identifying essential *M. mycetomatis* kinases not present in the human kinome

To identify essential kinases, the identified *M. mycetomatis* kinases were compared to the respective *A. nidulans* kinases, which have previously been evaluated by knock-out strategies [33]. Here, the sequences of kinase orthologues were identified by searching the respective proteins within the OrthoDB database and by protein-BLAST comparison of both the kinase domain as provided by Uniprot for the respective kinases, and the full protein with *A. nidulans* kinases [30,33–35]. It was assumed that when the *A. nidulans* kinase was found to be essential, than the *M. mycetomatis* orthologue was essential too.

### *In vitro* gene expression of the predicted kinases

*M. mycetomatis* strain MM55 was grown on Sabouraud Dextrose Agar (SDA, Cat. No. 210950, Becton Dickinson, Vianen, The Netherlands) at 37°C prior to experimental use. After two weeks of growth, the mycelium was harvested and transferred to RPMI (Cat. No. 11564456, Thermo Fisher Scientific, Breda, The Netherlands) supplemented with 0.35 g/L L-glutamine (Cat. No. BE17-605E, Lonza, Breda, The Netherlands) and 1.98 mM 4-morpholinepropanesulfonic acid (MOPS, Cat. No. M1254, Sigma Aldrich, Zwijndrecht, The Netherlands). The mycelium was sonicated at 10 µm for 10 seconds (Soniprep 150 plus, MSE, Heathfield, United Kingdom) to generate a fragmented hyphal suspension and incubated at 37°C [36]. After seven days the mycelium was harvested by centrifugation, washed, resuspended and sonicated as described above. A standardized hyphal suspension of 70% ± 2% transmission was prepared at 660 nm (Novaspec II; Pharmacia Biotech, Sweden) in RPMI medium supplemented with 0.35 g/L L-glutamine, 1.98 mM 4-morpholinepropanesulfonic acid (MOPS) and 1% DMSO (8.02912.1000, Merck, Schiphol-Rijk, The Netherlands). Hyphal suspensions were incubated at 37°C and after 4, 24 and 72 hours RNA was isolated according to a TRIzol-based method. In short, the cultures were centrifuged, the media was removed, and the biomass was suspended in TRIzol reagent (Cat. No. 15596018, Invitrogen, Bleiswijk, Netherlands) along with 5 metal beads (Cat. No. KM2000.003000.0010, DIT Holland B.V.). The samples were lysed using a tissue lyser (Qiagen, Hilden, Germany) for 150 seconds at 30 Hz. Following this, the disrupted sample was centrifuged at 12,000 RPM for 5 minutes at 4°C, and the supernatant without cell debris was transferred to a new Eppendorf tube. Chloroform (Cat. No. 34854, Sigma-Aldrich, Zwijndrecht, The Netherlands) was added to the samples, which were vortexed for 10 seconds and incubated at room temperature (21°C). After 5 minutes, samples were centrifuged at 12,000 RPM for 5 minutes at 4°C. The resulting upper phase was isolated without disturbing the interphase, and 1/10 volume of 3 M sodium acetate pH 5.5 (Cat. No. S7899, Sigma-Aldrich, Zwijndrecht, The Netherlands) and 1 volume of isopropanol (Cat. No. 1,09634.1000, Merck, Schiphol-Rijk, The Netherlands) were added. After careful mixing, the sample was incubated at room temperature for 20 minutes and centrifuged at 12,000 RPM for 5 minutes at 4°C. The supernatant was discarded, and the RNA pellet was washed twice with ice-cold 70% ethanol, followed by centrifugation at 12,000 RPM for 5 minutes at 4°C. After discarding the supernatant, the remaining pellet was air-dried for 15 minutes and then resuspended in diethyl pyrocarbonate (DEPC, Cat. No. D5758, Sigma-Aldrich, Zwijndrecht, The Netherlands)-treated water. The concentration and quality of RNA were assessed using Nanodrop One (Thermo Fisher Scientific, Breda, The Netherlands), and the samples were stored at -80°C for further processing.

RNA-seq library was prepared using MGIEasy RNA Directional Library Prep Set (Cat. No., MGI Tech, Shenzhen, China) according to the manufacturer’s protocols. We used 0.34 to 1 µg of total RNA measured by NanoDrop2000 (Thermo Fisher Scientific, Breda, The Netherlands) for each sample for the library preparation. PolyA + RNA was enriched from the total RNA with oligo dT beads prior to prepare the RNA-Seq library. The resulting RNA library was QCed using Agilent Bioanalyzer with High Sensitivity DNA assay kit (Cat. No. 5067–4626, Agilent Technologies, Santa Clara, California) and sequenced with 100 bp paired-end reads using a DNBSEQ G400 Sequencing platform (MGI Tech, Shenzhen, China) and a DNBSEQ-T7 Sequencing platform (MGI Tech, Shenzhen, China). After base calling of the raw reads from RNA-seq, FastQC (https://www.bioinformatics.babraham.ac.uk/projects/fastqc/) and seqKit_stat were used as standard QC methods [37]. STAR aligner was used for mapping the reads against the published genome of the *M. mycetomatis* [29]. The mapped reads in BAM format, were quantified using the Linux Subread package and featureCount command [38].

### *In vivo* gene expression of the predicted kinases

To prioritize the kinases identified as drug targets, we followed the predicted kinase encoding gene expression over time in an *in vivo Galleria mellonella* grain model. *G. mellonella* larvae from SAGIP were obtained from the Forelshop (Tremelo, Belgium). Prior to use, the larvae were housed in darkness on wood shavings at room temperature (21°C). Within five days of arrival, larvae weighing approximately 300–500 mg were selected and evenly distributed, five larvae per dish, across Petri dishes containing 90 mm Whatman filter paper. The inoculum was prepared and *G. mellonella* larvae were infected as previously described [22]. In short, *M. mycetomatis* MM55 mycelium was harvested after three weeks of growth on SDA at 37°C, transferred to RPMI supplemented with 0.35 g/L L-glutamine and 1.98 mM 4-morpholinepropanesulfonic acid. The mycelium suspension was sonicated at 10 µm for 30 seconds (Soniprep 150 plus, MSE) and incubated at 37°C. After two weeks the mycelium was harvested by vacuum filtration (Cat. No. 430758, Corning, Amsterdam, The Netherlands), washed twice with PBS, sonicated at 10 µm for 120 seconds, and resuspended in PBS to a final concentration of 100 mg fungal biomass/mL. The infection was induced by injecting the larvae with the prepared inoculum of *M. mycetomatis* strain MM55, to achieve a final concentration of 4 mg fungal biomass/mL. The injection was administered into the last left proleg using a 29G U-100 insulin needle (Cat. No. 324824, Becton Dickinson, Vianen, The Netherlands). After 6, 24, 72, and 168 hours post-infection, RNA was extracted by combining the contents of three larvae while excluding the intestine, swiftly freezing the contents with liquid nitrogen, and pulverizing the frozen material using a pestle and mortar. The resulting powder was then suspended in RLT buffer supplemented with 1% β-mercaptoethanol (Cat. No. M7522, Sigma-Aldrich, Zwijndrecht, The Netherlands), as provided in the RNeasy Mini Kit (Cat. No. 74104, Qiagen, Hilden, Germany). Subsequently, the samples were incubated at 57°C for 3 minutes before proceeding in accordance with the manufacturer’s instructions. The concentration and quality of RNA were assessed using Nanodrop One, and the samples were stored at -80°C for further processing.

RNA-Seq libraries (n = 15) were prepared using 1 µg of total RNA with TruSeq Stranded mRNA Library Prep kit (Cat. No. 20020595, Illumina, San Diego, California) following the manufacturer’s instructions. The HiSeq2500 instrument (Illumina, San Diego, California) was used for sequencing with Paired-End; 100base. The quality of the raw reads was checked with FastQC (https://www.bioinformatics.babraham.ac.uk/projects/fastqc/). RNA-seq library QC of the sequenced tags per sequence library was performed. Using STAR Aligner [39], the *G. mellonella* larvae fastq files were mapped twice, first against *G. mellonella* assembly ASM364042v2 and then against *M. mycetomatis* genome assembly ASM127576v2. The mapping of the raw sequence reads was QCed. Details of the genome-wide whole-transcriptome analysis in [40]. The STRING database was used for predicting the Protein-Protein Interaction (PPI) network [41]. We used the list of the genes involved in the WCI biosynthesis pathways in Fig 4A (8 genes) and performed the search again the *Madurella mycetomatis* species. We used the following as basic settings parameters. Network type as full STRING network, meaning of network edges as evidence, active interaction sources (Textmining, Experiments, Databases, Co expression, Neighborhood, Gene Fusion, and Co-occurrence). We used minimum required interaction score = 0.400 (medium confidence) and max number of interactors to show: (query proteins only). The resulting PPI in Fig 4a have the following characteristics: number of nodes: 8, number of edges: 17, average node degree: 4.25, avg. local clustering coefficient: 0.775, expected number of edges: 2, and PPI enrichment p-value: 1.48e-10.

**Fig 2:**
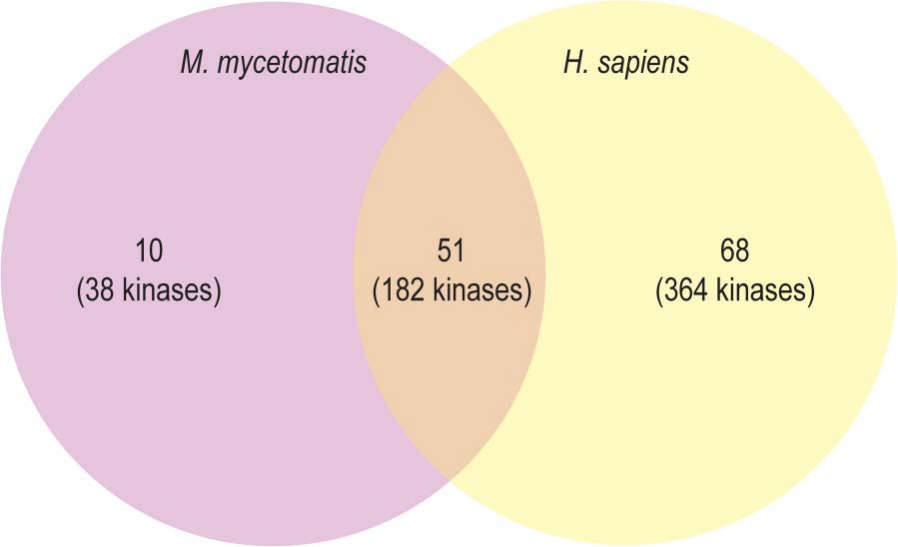
Kinome cluster comparison between *Homo sapiens* and *M. mycetomatis.* Cluster analysis OrthoVenn3 as shown in a Venn diagram. The clusters are indicated by numbers and the kinases within each cluster are indicated between brackets. Orthologue analysis identified 10 clusters containing 38 kinases unique to *M. mycetomatis*, 68 clusters containing 364 kinases unique to *H. sapiens*, and 51 cluster containing 182 kinases of both *M. mycetomatis* and *H. sapiens*.

**Fig 3:**
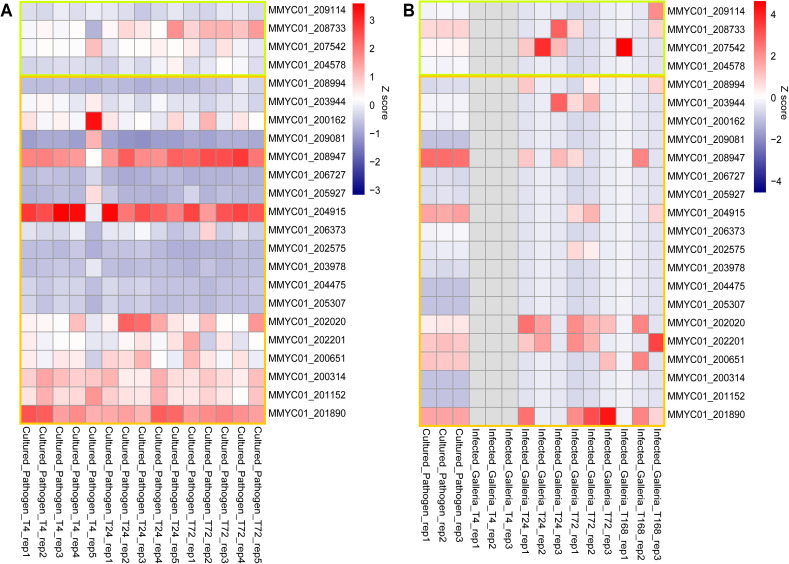
Expression of essential kinase genes. In the heatmap, the X-axis represents the samples, and the Y-axis represent *M. mycetomatis* essential kinase set of genes using a Z-scale. Genes encoding for kinases grouped in category 1 are highlighted in the green box, and genes encoding for kinases grouped in category 2 are highlighted in yellow. A. Heatmap of the expression of the essential kinases genes detected in the transcriptomics analysis of the *in vitro* experiments. *M. mycetomatis* pathogen cultured in 1% DMSO and five replicates are collected at 3 time points. B. Heatmap of the expression of the essential kinases set of genes detected in the transcriptomics analysis of the *in vivo* experiments. X-axis depict the different samples at the different time points, namely *M. mycetomatis* cultured pathogen (T = 0 hours), and the *Galleria mellonella* larvae infected by *M. mycetomatis* at T = 4 hours, T = 24 hours, T = 72 hours, and T = 168 hours. Data source was provided as DataSource_MainFig3A and DataSource_MainFig3B.

### Protein structure prediction

The homology modeling of the proteins encoded by MMYC01_208733 and MMYC01_207542, was carried out using AlphaFold (https://alphafoldserver.com/) due to the unavailability of experimental models [42]. The predicted three-dimensional structure of proteins were based on their amino acid sequences. The ERRAT and PROCHECK Ramachandran plot analyses were performed to assess the quality and stereochemical validation of the protein structure [43,44]. ERRAT, a structure validation tool, evaluates the overall quality of the model by analyzing non-bonded atomic interactions, generating an error function-based quality score. Values above 90% indicate a reliable structure. The protein model in Protein Data Bank (PDB) format was uploaded to the ERRAT server for evaluation. PROCHECK was used to analyze the backbone dihedral angles (ϕ and ψ) through the Ramachandran plot, assessing stereochemical quality by categorizing residues into favored, allowed, and disallowed regions. The model was uploaded to the PROCHECK server, and the plot was generated to verify conformational integrity, ensuring the structure adhered to standard stereochemical parameters. These analyses provided critical insights into the reliability and accuracy of the modeled protein structure.

### Molecular docking

The target protein refinement was performed using CHIMERA V1.16 [45]. Standard residues present in the protein were minimized using the AMBER force field, while non-standard residues were minimized using AM1-BCC semi-empirical charges to maintain proper electrostatic interactions. Non-essential residues, such as water, co-crystal ligands, and unnecessary chains, were removed, as they may interfere with the docking procedure. The structures of the ligands were drawn in Marvin Sketch (http://www.chemaxon.com/), and hydrogens were added. Optimal 2D and 3D clean configurations were selected, and possible conformers were generated from which the lowest-energy conformer was chosen and saved in MOL2 format. Then, the structures were optimized using CHIMERA with the AM1-BCC force field. Proteins and ligands were converted to PDBQT format using AutoDockTools, which can be read by the Vina tool [46]. In AutoDockTools, the rotatable bonds in the ligands were identified and made flexible during the docking study for conformational variability. The protein was kept rigid, while the ligand’s flexibility was explicitly considered using flexible rotatable bonds. It is important to note that docking was performed without explicit solvent conditions. Active site amino acids were identified using the CASTp server [47]. Then the Grid box was defined around the active site, and the grid dimensions were optimized to encompass the protein-ligand interactions. We used Autodock Vina V. 1.2.6 for molecular docking [48,49]. The docking parameters were optimized by multiple docking runs using various grid sizes and based on different tools to identify the best binding pose and replicable results like spacing (0.375 Å), num_modes (9), energy range (3), exhaustiveness (16), and other default configurations. Docking results were visualized through Biovia Discovery Studio visualization tool (https://www.3ds.com/products-services/discovery-studio/) and Maestro 12.3 (Academic Edition) (https://www.schrodinger.com/maestro) for structural analysis, and binding interactions were noted in the table.

### Screening the kinase chemogenomic set

The KCGS library was screened according a previously described method for *in vitro* susceptibility testing for *M. mycetomatis* using luciferase [50]. In short, a standardized hyphal suspension was prepared as described above and 54.4 µL volume was transferred to a white 384 wells-plate (Cat. No. 781080, Greiner Bio-One, Alphen aan de Rijn, Netherlands) containing 0.6 µL of the respective kinase inhibitor and mixed by resuspending 30 times. DMSO (solvent) was used as growth control, and RPMI media without fungal hyphae as a negative control. The plate was sealed to prevent evaporation and incubated for 4 days at 37°C under 5% CO_2_ conditions. After incubation 30 µL of luciferase from the CellTiter-Glo 3D Cell viability assay (Cat. No. G9683, Promega, Leiden, The Netherlands) was added to the respective wells and mixed by resuspending 30 times. After 25 minutes of incubation at room temperature (21°C), luminescence was read using the CytoFluor Series 4000 (PerSeptive Biosystems, Framingham, USA). The resulting luminescence output was used to calculate metabolic activity as a marker for fungal growth according to the formula:


$$Metabolic\;activity\;=\;\frac{\left(Luminescence\;Sample-Luminescence\;Negative\;Control\right)}{\left(Luminescence\;Growth\;Control\;-\;Luminescence\;Negative\;Control\right)}*100\;\%$$

The library was primarily screened at 1, 2 and 10 µM by performing two biological replicates. True positives, defined as hits in both biological replicates based on a reduction of ≥ 80% in metabolic activity, were also screened at 4 and 8 µM. Finally, to determine the IC_50_, the calculated growth was plotted against the respective drug concentrations. The resulting graphs were used to visually determine the IC_50_ for each compound. The IC_50_ was determined in duplicate and the means plus standard deviations were determined in Excel.

### Screening MycetOS compounds

The MycetOS compounds were screened according to the described method for *in vitro* susceptibility testing and the drug screening pipeline established within the MycetOS project [25]. In short, the compounds were transferred to Costar 96-well round bottom plates (Cat. No. 3799, Corning, Amsterdam, The Netherlands) and further diluted in standardized hyphal suspension, prepared as described above, to final concentrations of 100 and 25 µM in 1% DMSO. The cultures were incubated for 7 days at 37°C under 5% CO_2_ conditions. After incubation, MTS (Cat. No. G3581; Promega, Leiden, The Netherlands) was added to each well to final concentration of 158.5 µg/mL. The plate was further incubated for another 20 minutes at 37°C before transferring the supernatant to flat bottom plates (Cat. No. 655101, Greiner Bio-One, Alphen aan de Rijn, Netherlands). The absorbance of the supernatant was determined spectrophotometrically at 490 nm using the EPOCH2 microplate reader (BioTek, Santa Clara, USA), and fungal growth was calculated using metabolic activity as a marker according to the formula below.


$$Metabolic\;activity\;=\;\frac{\left(Absorbance\;Sample-Absorbance\;Negative\;Control\right)}{\left(Absorbance\;Positive\;Control\;-\;Absorbance\;Negative\;Control\right)}*100\%$$

In this formula the positive control is the absorbance measured after growing the fungus in RPMI with solvent (1% DMSO) only. The negative control is the absorbance measured after incubating the culture media with solvent but without fungal hyphae.

Compounds were screened in triplicate and inhibition of fungal growth was defined as a reduction of ≥ 80% in metabolic activity. Compounds that can inhibit fungal growth at 25 µM were further screened in a dilution series ranging from 0.03 to 16 µM and the IC_50_ was calculated as described above.

## Results

### Kinome of *M. mycetomatis*

Kinannote identified in total 159 proteins of interest in the proteome of *M. mycetomatis* (UP000078237). Among these proteins, 108 eukaryotic protein kinases and 4 atypical protein kinases were identified. Furthermore, 24 proteins were classified as “twilight”, indicating these are protein kinase subdomain-containing proteins by the Kinannote program. Manual curation of these 24 proteins based on sequence homology with functionally annotated kinases of *A. nidulans* and *N. crassa*, as well as InterPro domain analysis identified 17 of these 24 proteins as predicted kinases. The Kinannote program reported 23 proteins as subthreshold, indicating these candidate proteins were rejected as protein kinases candidates. Overviews of the group classification based on Kinannote and the complete curated overview of all identified 132 predicted kinases are listed in S1 and S2 Tables respectively.

### Common and essential proteins in *M. mycetomatis*

OrthoVenn3 revealed 51 clusters containing a total of 182 kinases which were predicted as orthologues between the kinome of *M. mycetomatis* and *H. sapiens*. From the 132 identified *M. mycetomatis* kinases, 52 kinases had predicted orthologues in the kinome of *H. sapiens*. Furthermore, the analysis revealed 10 clusters containing 38 kinases unique to *M. mycetomatis* and 42 proteins were identified as singletons (Fig 2). The 10 unique clusters consist of 38 *M. mycetomatis* kinases which do not share orthologues with human kinase proteins (S3 Table). Further analysis of the 42 singletons using OrthoDB identified 30 proteins which do not share orthologues with human kinases. To summarize, 68 *M. mycetomatis* kinases did not have orthologues with the human kinome (S2 Table).

From the 132 identified *M. mycetomatis* kinases, OrthoDB in combination with protein-BLAST revealed 96 orthologue kinases in both *A. nidulans* and *N. crassa.* Of the identified kinases, based on shared homology with *A. nidulans* and the conserved nature of kinase proteins throughout eukaryotic lifeforms, 21 are predicted as essential kinases for fungal viability [28,51]. We therefore assumed that these identified kinases are likely essential for *M. mycetomatis* as well. Full functional annotation of the kinases gene was provided in S4 Table and S1 Fig.

The complete overview of shared orthologues and corresponding phenotypic analysis of the *A. nidulans* orthologues is shown in S2 Table.

Based on the common and essential proteins as predicted according by the above method, the kinome of *M. mycetomatis* was divided into four categories based on the potential as a drug target. The first category consists of the kinases without orthologues in the human kinome that were predicted as essential for fungal viability. The second category includes kinases that were predicted as essential for fungal viability but had orthologues in the human kinome. The third category consists of kinases unique to *M. mycetomatis*, but which were not predicted as essential for fungal viability. Finally, the fourth category consists of kinases with orthologues in the human kinome which were not predicted as essential for fungal viability. The complete overview of the categorized kinases is visualized in S5 Table. The kinases identified in category 1 were predicted to be essential and fungal-specific, and therefore were deemed most promising as drug targets. This category consists of four kinases, namely cytokinesis protein sepH (MMYC01_209114), MAP kinase kinase Pek1/Mkk2 (MMYC01_208733), MAP kinase kinase kinase mkh1 (MMYC01_207542), Serine/threonine-protein kinase nak1 (MMYC01_204578). These kinases were classified as belonging to the STE group of kinases, a group based on homology to yeast proteins STE20, STE11, and STE7 which result in a STErile phenotype if deleted [52].

### Expression of *M. mycetomatis* kinase encoding genes

**Fig 4:**
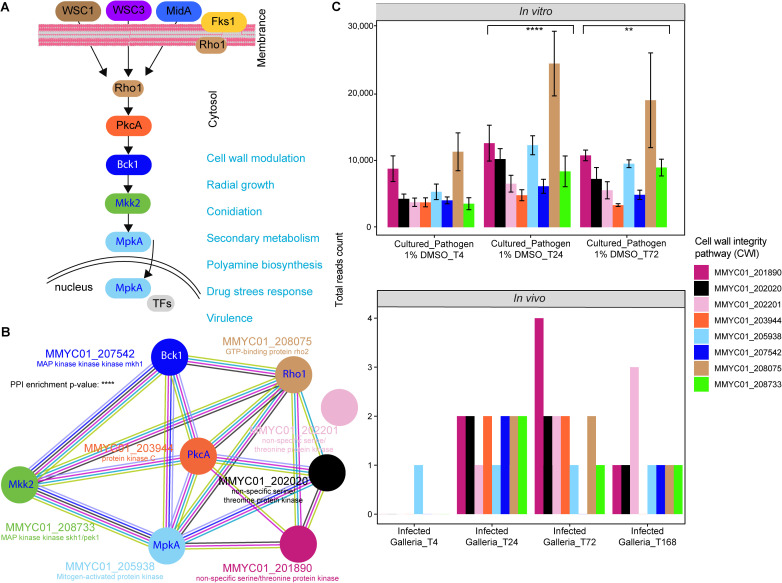
Analysis of the Cell Wall Interaction (CWI) pathway in *M. mycetomatis.* A. CWI signaling pathway in *A. fumigatus* re-drawn from Valiante et al. [85]. The schematic representation shows five main genes involved in the CWI pathway and their function. B. Protein-Protein-Interaction (PPI) network of the CWI pathway in *A. nidulans* and *M mycetomatis*, and the relation to the additional three essential *M. mycetomatis* genes with relatively high *in vivo* expression. Each node represents a gene, and the edge in the network represents the predicted functional associations. The color codes for each edge explained in the STRING database [41]. The PPI enrichment p-value is 9.46e-08 for *M. mycetomatis* proteins. C. Upper panel shows the expression of the CWI genes from *in vitro* analysis. The values in the bar chart represents the mean of the reads count and the bar represent the SEM. The Wilcoxon test compared time points T = 24 hours & T = 4 hours, and T = 72 hours & T = 4 hours after infection. The bottom panel shows the expression of the CWI genes from *in vivo* transcriptomics analysis. Data source was provided as DataSource_MainFig4C_InVitro and DataSource_MainFig4C_InVivo.

To prioritize the kinases as potential drug targets, the expression of the essential kinases was determined during fungal growth *in vitro* in RPMI and during *in vivo* infection in *G. mellonella*. We returned 245,960,792 total tags for the *G. mellonella* larvae samples and 152,647,712 total tags for the *M. mycetomatis* samples. A summary of the total tags, and the overview of mapped and unmapped reads is provided in S6 and S7 Tables, respectively. The full overview of all kinase expression levels is provided in S5 Table for the *in vitro* and *in vivo* data, and visualized in S2 and S3 Figs respectively. Fig 3 shows the *in vitro* and *in vivo* expression for the 23 genes coding for the 21 essential kinases (categories 1 and 2). Not all kinases in these categories were expressed *in vitro* during growth in optimal conditions, or detected during infection in *G. mellonella*. Of the four promising essential kinases in category 1, two (MMYC01_209114 and MMYC01_204578) were not expressed *in vitro* and *in vivo*. On the other hand, MMYC01_208733 and MMYC01_207542 were expressed during infection, namely at 24h post-infection (MMYC01_208733 and MMYC01_207542), at 72h post-infection (MMYC01_208733) and at 168h post-infection (MMYC01_208733 and MMYC01_207542). These two genes, encoding for *M. mycetomatis* orthologues of Mkh1/Bck1 and Pek1/Mkk2, are part of the Cell Wall Integrity (CWI) pathway, as well as MMYC01_203944 (orthologue of Pck2/PkcA) which is also expressed during infection (Fig 4A) [53]. To confirm the interaction between the different *M. mycetomatis* genes related to the CWI pathway, a Protein-Protein Interaction (PPI) network of the proteins related to the CWI pathway in *M. mycetomatis* was constructed, indicating the predicted interaction between the different proteins (Fig 4B). Further analysis of this pathway in Fig 4C, revealed that the entire pathway was expressed *in vitro* and expression of fungal transcripts was detected in *G. mellonella.*

Other kinases with relatively high expression *in vivo* are MMYC01_202020, MMYC01_202201 and MMYC01_201890, *M. mycetomatis* orthologues of Ksg1, Prp4, Tor2 (TorA in *A. nidulans*) in *Schizosaccharomyces pombe* respectively. The PPI analysis revealed predicted protein interactions between MMYCO_202020 and MMYC01_201890 with the CWI pathway (Fig 4B). Based on the same PPI analysis in Fig 4B, MMYC01_202201 had no predicted interaction with the CWI pathway. We further look at the enrichment of the PPI nodes. The associated GO terms with the PPI and their statistical significance is shown in S1 Fig. (PPI GO TERSM).

### Docking of MMYC01_208733 and MMYC01_207542 identified several kinase inhibitors with high affinity for these kinases

**Fig 5:**
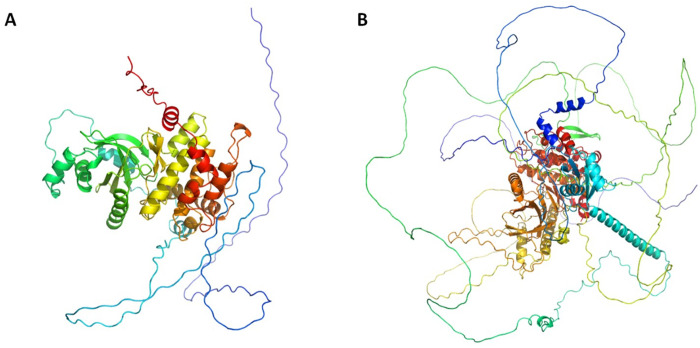
Structure of the modelled kinases encoded by MMYC01_208733 and MMYC01_207542. A. modelled MAP kinase mkh1 encoded by MMYC01_208733. B modeled MAP kinase skh1 encoded by MMYC01_207542.

**Fig 6:**
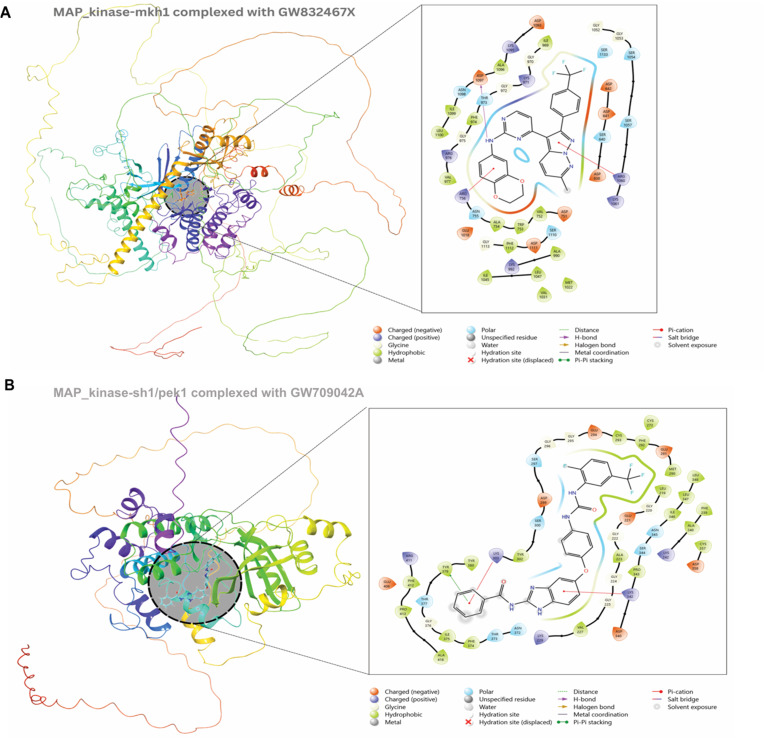
3D and 2D protein-ligand interaction of MAP_kinase_mkh1 with GW709042A, and MAP_kinase_skh1 with GW832467X.

To be able to identify potential inhibitors active on MMYC01_208733 and MMYC01_207542, their protein structures were predicted using AlphaFold (Fig 5) (S1 Data). Molecular docking-based virtual screening was carried out for 308 kinase inhibitors derived from the KCGS small molecule library and the MycetOS project against MMYC01_208733 and MMYC01_207542. The docking results were analyzed in terms of binding energy, affinity, and the type of interactions at the active site of the selected targets (Fig 6) (S8 Table). For MMYC01_208733 the 10 compounds with lowest binding energy are listed in Table 1.

**Table 1:** The top 10 hits with the lowest binding energy for MMYC01_208733 and MMYC01_207542.

Target	Compound	SMILES	Binding energy (kcal/mol)
MMYC01_208733	GW709042A	Cl.Fc1ccc(cc1NC(=O)Nc1ccc(Oc2ccc3[nH]c(NC(=O)c4ccccc4)nc3c2)cc1)C(F)(F)F	-11.5
	GW683134A	Cl.Fc1ccc(cc1NC(=O)Nc1ccc(Oc2ccc3[nH]c(NC(=O)c4ccco4)nc3c2)cc1)C(F)(F)F	-11.4
	GSK312879	Fc1cccc(F)c1C(=O)Nc1cccc(c1)-c1nn2ncccc2c1-c1ccnc(Nc2cccnc2)n1	-11.3
	GW830263A	Cl.CN(c1ccc(NC(=O)Nc2cccc(c2)C(=O)N2CCN(C)CC2)cc1)c1ccnc(Nc2ccc(CS(C)(=O)=O)cc2)n1	-11.3
	TX1-85-1	CC(=O)N1CCN(CC1)C2CCC(CC2)N3C4=C(C(=N3)C5 = CC(=C(C = C5)OC6 = CC = CC = C6)NC(=O)C = C)C(=NC = N4)N	-11.2
	DDR1-in-1	CC1 = C(OC4 = CC(CC(N5)=O)=C5C=C4)C = C(NC(C2 = CC = C(CN3CCN(CC)CC3)C(C(F)(F)F)=C2)=O)C = C1.Cl.Cl	-11.1
	AKI-014a	ClC6(=C(N4(C(=O)C3(=C(N=C(NC2(=CC=C(N1(CCCCC1))C = C2))N = C3)N5(C4 = NC = C5))))C = CC = C6).FC(F)(F)C(=O)O	-10.9
	D2202-1	Cc1cn(cn1)c1cc(cc(c1)NC(c1ccc2c(CN(C[C@@H]2C)c2cncnc2)c1)=O)C(F)(F)F	-10.9
	MRKI-24	Cc1ccc(NC(=O)c2cccc(c2)C3(CCOCC3)C#N)cc1C(=O)Nc4ccc5ncsc5c4	-10.9
	GSK1838705	CNC(C1 = C(NC2 = NC(NC3 = C(OC)C = C4CCN(C(CN(C)C)=O)C4 = C3)=NC5 = C2C=CN5)C = CC = C1F)=O	-10.8
MMYC01_207542	GW832467X	Cc1ccc2c(c(nn2n1)-c1ccc(cc1)C(F)(F)F)-c1ccnc(Nc2ccc3OCCOc3c2)n1	-11.4
	GSK312879	Fc1cccc(F)c1C(=O)Nc1cccc(c1)-c1nn2ncccc2c1-c1ccnc(Nc2cccnc2)n1	-10.3
	AKI-001a	FC5(=C(C = 1(N = C4(CCCN4(C = 1C3(=NN2(C(=NN = C2C(O)(C)C)C = C3))))))C = CC(=C5)F)	-10.0
	GW807982X	CCOc1ccc2c(cnn2n1)-c1ccnc(Nc2cc(OC)cc(c2)C(F)(F)F)n1	-9.8
	GW811761X	CCOc1ccc2c(cnn2n1)-c1ccnc(Nc2cccc(c2)C(F)(F)F)n1	-9.8
	GW683134A	Cl.Fc1ccc(cc1NC(=O)Nc1ccc(Oc2ccc3[nH]c(NC(=O)c4ccco4)nc3c2)cc1)C(F)(F)F	-9.8
	GSK1379710	CC(=O)c1cccc(NC(=O)NC2CCN(C2)c2ccnc(Nc3ccc(F)cc3)n2)c1	-9.7
	GW810372X	COc1ccc2c(cnn2n1)-c1ccnc(Nc2cccc(OC(F)(F)F)c2)n1	-9.7
	UNC-CAF-061	CCOC1 = NN2N=CC(C3 = NC(NC4 = CC = CC(OC(F)(F)F)=C4)=NC = C3)=C2C=C1	-9.7
	SGC-CLK-1	COC1 = NN2N=CC(C3 = NC(NC4 = CC(C(F)(F)F)=CC(OC)=C4)=NC = C3)=C2C=C1	-9.7

### Library screen identifies eight kinase inhibitors that inhibit *M. mycetomatis*

**Fig 7:**
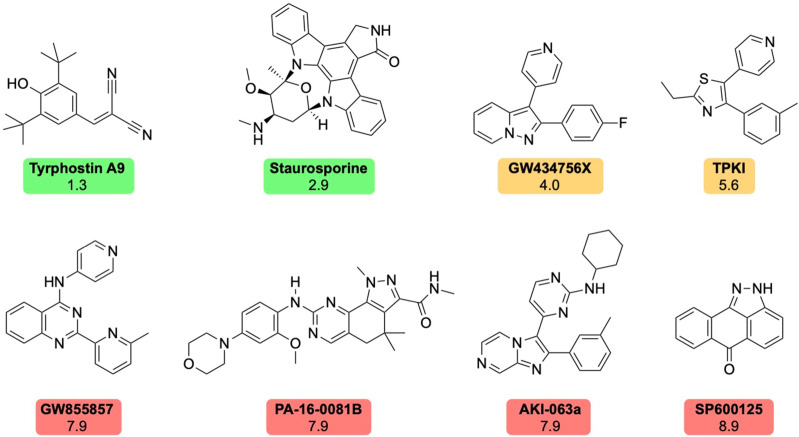
The molecule structures of the eight compounds identified from the *in vitro* screening of the KCGS library and MycetOS compounds which inhibiting the growth of *M. mycetomatis.* The name of each compound is indicated under the molecule structure. Under the compound name the IC50 in µM is given. The compounds with an IC50 lower than 3.0 were indicated in green. Compounds with an IC50 between 3.0 and 6.0 µM were indicated in orange. And compounds with an IC50 higher than 6.0 µM were indicated in red.

After demonstrating with the docking studies that several kinase inhibitors had a high affinity for the two fungal specific targets, we determined the *in vitro* activity of the 308 kinase inhibitors derived from the KCGS small molecule library and the MycetOS project against *M. mycetomatis.* A full overview of the KCGS and MycetOS screening results is provided in S8 and S9 Tables. Screening the KCGS library and MycetOS compounds identified eight compounds that were capable of inhibiting the growth of *M. mycetomatis* as shown in Table 2, S9 Table and Fig 7. Tyrphostin A9 was the compound with the lowest IC_50_ concentration (1.3 µM) which is comparable with its recently disclosed activity against *Coccidiodes posadassii* (IC50 = 0.9 µM) [54]. Based on the docking studies, tyrphostin A9 was able to interact with MMYC01_207542 via hydrophobic interactions between LEU219A and VAL277A and tyrphostin A9 and hydrogen bonds between SER300A, SER344A and ASP358A and tyrphostin A9 (S4 Fig). Predicted hydrophobic interactions between tyrphostin A9 and residue TRP753A of MMYC01_208733 and hydrogen bonds between SER1054A, ARG1060A, ASP1097A and ASP1111A were also predicted. Since, all eight kinase inhibitors exhibit binding affinity with multiple human kinases, we also postulated that these kinase inhibitors might have multiple targets in *M. mycetomatis* too [55]. Based on orthologue identification of known drug targets using OrthoDB, *M. mycetomatis* homologues were identified (Table 2). For tyrphostin A9, no distinct orthologues are defined among these listed targets based on OrthoDB identification. Based on protein-BLAST the most similar *M. mycetomatis* protein was the predicted essential and unique kinase MMYC01_209114 (KXX73803.1, 32% protein similarity, 80/250 amino acid), suggesting non-selective targeting of Tyrphostin A9 [56].

**Table 2:** The top 8 hits from the KCGS library and MycetOS compounds with corresponding targets and IC_50_ values. *M. mycetomatis* orthologues were identified based on OrthoDB and protein-BLAST analysis. Highlighted orthologues in *M. mycetomatis* are linked to the CWI pathway. Notably, kinases likely exhibit more off-targets than listed below [86].

Compound	SMILES	Chemical class	Target(s)	M. mycetomatis orthologues	Binding energy (kcal/mol) against MMYC01_208733	Binding energy (kcal/mol) against MMYC01_207542	IC50 (µM)	Stoplight score (https://stoplight.mml.unc.edu/)
GW855857	Cc1cccc(n1)-c1nc(Nc2ccncc2)c2ccccc2n1	pyridylamino-substituted quinazoline derivative	ACVR1B, PRKD1, PRKD2, PRKD3, TGFBR1	No clearly defined orthologues for the listed drug targets.	-8.721	-9.084	7.94	0.24
TPKI-106	CCC1 = NC(=C(S1)C1 = CC = NC = C1)C1 = CC(C)=CC = C1	pyridyl-substituted thiazole derivative	MAPK10, MAPK14	MMYC01_204641, **MMYC01_205938**	-7.368	-7.76	5.58	0.35
GW434756X	Fc1ccc(cc1)-c1nn2ccccc2c1-c1ccncc1	pyridyl-substituted pyrazolopyridine derivative	CSNK1A1, MAPK10, MAPK14, MAPK9, NLK, RPS6KA6	MMYC01_208878, MMYC01_204915, MMYC01_204641, **MMYC01_205938**	-8.072	-8.053	3.99	0.35
AKI-063a	N1(=C(N = CC = C1C=3(N2(C(C = NC = C2)=NC = 3C4(=CC(=CC=C4)C))))NC5(CCCCC5))	pyrimidyl-substituted imidazopyrazine derivative	MAPK11, MAPK10, MAPK9, MAPK8, MAPK14, RPS6KA6, GAK, RPS6KA1	MMYC01_203878, MMYC01_204641, **MMYC01_205938**	-9.498	-7.787	7.91	
PA-16-0081B	O = C(NC)C1 = NN(C2 = C1C(C)(CC3 = CN = C(N = C23)N([H])C4 = C(C = C(C = C4)N5CCOCC5)OC)C)C	dihydropyrazoloquinazoline derivative	DYRK1B, CLK2, CLK1, DYRK2*, GAK, DYRK1A	MMYC01_206522, MMYC01_202450, MMYC01_201890, MMYC01_203320, MMYC01_203321, MMYC01_200523, MMYC01_207837, MMYC01_202192, MMYC01_208092	-9.739	-7.221	7.86	
Tyrphostin A9	N#C/C(C#N)=C/C1 = CC(C(C)(C)C)=C(O)C(C(C)(C)C)=C1	3,5-di-*tert*-butyl-4-hydroxybenzylidene-malononitrile	PYK2, PDGF, and VEGF	MMYC01_209114	-7.049	-6.989	1.3	0.29
Staurosporine	CC12C(C(CC(O1)N3C4=CC = CC = C4C5=C6C(=C7C8=CC = CC = C8N2C7=C53)CNC6 = O)NC)OC	natural indolocarbazole	PKC, PKA, c-Fgr, and phosphorylase kinase	**MMYC01_203944**, MMYC01_205810, MMYC01_202629, MMYC01_203718, MMYC01_205718, MMYC01_206408, MMYC01_205807, MMYC01_209081, MMYC01_208365, MMYC01_210358	-7.539	-6.11	2.9	0.35
SP600125	O = C1C2=C3C(NN = C3C4=C1C = CC = C4)=CC = C2	1,9-pyrazoloanthrone	Mapk8, Mapk9, and Mapk10	MMYC01_204641 and **MMYC01_205938**	-8.998	-7.866	8.9	0.29

Based on the orthologue identification with OrthoDB, TPKI-106, GW434756X, AKI-063a and SP600125 were predicted to have binding affinity with the MMYC01_205938 (Pmk1/MpkA), an orthologue that belongs to the CWI pathway. Staurosporine was predicted to have binding affinity with MMYC01_203944 (Pck2/PckA) of the CWI pathway.

## Discussion

In the *M. mycetomatis* genome we identified 132 predicted protein kinases, a number that is comparable to the 103, 107, and 106 predicted kinases in the proteomes of *Candida albicans*, *Candida parapsilosis* and *Candida tropicalis*, and the 131, and 137 predicted kinases and 107 predicted kinase genes in the genomes of *A. nidulans*, *Aspergillus fumigatus*, and *N. crassa*, respectively [18,33,57]. Kinases play critical roles in the response to osmotic stress, regulation of cell wall integrity and virulence in several pathogenic fungi, such as *Saccharomyces cerevisiae*, *C. albicans*, *Cryptococcus neoformans*, *A. nidulans* and *A. fumigatus* [19,33,58–60]. Due to these essential roles, fungal kinases are considered promising targets for antifungal drug development with potential for synergistic combinations [19,61,62].

Therefore, in this study we focussed on evaluating the kinome of *M. mycetomatis* to identify candidate kinases which are predicted as suitable drug targets. Based on orthologue comparison with the functional annotation of the *A. nidulans* kinome and the human kinome, 21 *M. mycetomatis* kinases were predicted to be essential for viability. Four of these kinases did not have orthologues in the human kinome and therefore considered ideal drug candidates. These four kinases are orthologues of Cdc7/SepH (MMYC01_209114), Mkh1/Bck2 (MMYC01_208733), Pek1/Mkk2 (MMYC01_207542), and Nak1/Pob6 (MMYC01_204578), and all belong to the STE group kinases thought to be involved in septation (Cdc7/SepH), cell growth and polarity (Nak1/Pob6), and in the CWI pathway (Mkh1/Bck2 and Pek1/Mkk2 in *S. pombe/A. fumigatus*) [53,63,64]. Cdc7/SepH and Nak1/Pob6 are not expressed *in vitro* or during grain formation in *G. mellonella* larvae. In contrast, Mkh1/Bck1, Pek1/Mkk2 and two additional orthologues within the CWI pathway (Pck2/PckA (MMYC01_203944) and Pmk1/MpkA (MMYC01_205938)), as well as MMYCO_202020, MMYC01_202201 and MMYC01_201890, are expressed during infection in *G. mellonella*. Although MMYCO_202020, MMYC01_202201 and MMYC01_201890 are not directly involved in the CWI pathway, MMYCO_202020 and MMYC01_201890 are predicted to interact with the CWI pathway. Previous research indicates that the respective orthologues Ksg1 in *S. pombe* and *A. nidulans* (AN3110), and Tor2/TorA, in *A. nidulans* (AN4936), are linked to the CWI pathway by inducing Pck2 mediated activation of the pathway in response to cellular stress such as oxidative stress [65,66].

While expression of the CWI-related genes was not observed in all samples, the significance of this finding is emphasized by the experimental conditions. First of all, as expected, in the *in vivo* transcriptome samples, the *G. mellonella* transcripts were more abundant than the *M. mycetomatis* transcripts. Due to this discrepancy in abundance between *G. mellonella* and *M. mycetomatis* transcripts, a relatively low number of fungal transcripts was detected in our sample set and especially for MMYC01_208733 this resulted in variation in expression levels between the replicates. On the other hand, genes for which a single copy was detected, could be disproportionately represented based on a technical bias such as the sequencing method, transcript stability, or just coincidence. However, the experimental limitations suggest that in order to detect *M. mycetomatis* derived transcripts, these genes are expected to exhibit relatively high expression levels in these samples [67,68]. Despite these limitations in the experimental procedure, the data does suggest the potential of the CWI pathway as an interesting drug target for eumycetoma caused by *M. mycetomatis*.

The CWI pathway consists of multiple mitogen-activated protein kinases (MAPK) that form a signalling cascade by sequential phosphorylation of the respective kinases. Phosphorylated Pmk1/MpkA moves into the nucleus and activates transcriptional regulators, regulating cell wall synthesis, maintenance and cellular stress response [69–71]. In *A. fumigatus* it has been shown that Pmk1/MpkA is vital for fungal growth, and it has also been linked to pyomelanin production and siderophore production [72]. For *C. neoformans* Pmk1/MpkA mutants showed reduced virulence in a cryptococcosis mouse model, and Mhk1/Bck1, Pek1/Mkk2, and Pmk1/MpkA were required for thermal stress adaptation [73,74]. Functional analysis of the *Fusarium graminearum* kinome highlighted CWI orthologues of Mhk1/Bck1, Pek1/Mkk2, and Pck2/PkcA as essential for vegetative growth, and that Mhk1/Bck1 and Pek1/Mkk2 mutants were defective in plant infection [75]. Since there is no method for disrupting genes in *M. mycetomatis*, we could not verify if similar effects would be found when *M. mycetomatis* orthologues of these genes would be disrupted. This is part of our ongoing work. Despite this limitation, the significance of the CWI pathway in fungal growth and pathogenesis remains interesting as the cell wall is a distinct fungal feature that is absent in mammalian cells, therefore, interference of this pathway could serve as an excellent target for antifungal drug development and should be explored further [76].

As of 2024, 80 FDA approved kinase inhibitors are available, and predominantly used as remarkably effective anti-cancer therapies [77,78]. Unfortunately, most of the kinases found in the CWI pathway are fungal-specific, and therefore, until now, there are no specific kinase inhibitors developed exclusively to target these fungal kinases. However, from studies on human kinase inhibitors it was noted that several of these kinase inhibitors are not entirely selective and can inhibit multiple kinases [79]. Therefore, we first modelled a kinase inhibitor library *in silico* to determine which of these kinase inhibitors could bind to the selected targets and then screened the human kinase inhibitors available *in vitro* to determine if any of these inhibitors would be able to inhibit *M. mycetomatis* growth. *In silico,* several kinase inhibitors could bind to MMYC01_208733 and MMYC01_207542, however none of these compounds inhibited the growth of *M. mycetomatis.* A reason behind this could be that these identified compounds could not cross the fungal cell wall and/or membrane and therefore not reach MMYC01_208733 and MMYC01_207542 in the cell. Compounds TPKI-106, GW434756X, AKI-063a and SP600125 were able to inhibit fungal growth. Based on homology searches, these compounds were predicted to also be able to inhibit the CWI-linked Pmk1/MpkA kinase. Although this is not accounting for any potential off-target binding of these kinase inhibitors in the fungal cell, it does suggest the importance of MAP kinases and the CWI pathway, thus providing a starting point for further drug development efforts.

Another known non-selective PKC inhibitor able to inhibit *M. mycetomatis* growth was staurosporine [70]. In *C. albicans*, it has been reported that staurosporine promotes filamentation, but causes defects in cell cycle progression and mislocalization of septin rings [80]. While staurosporine has been postulated to affect the CWI pathway, pinpointing the mechanism by which cellular processes are obstructed by staurosporine is challenging since it is a non-selective inhibitor. In *S. cerevisiae*, it was shown that staurosporine decreases Pmk1/MpkA phosphorylation, suggesting that staurosporine does affect the CWI pathway [81]. Similar findings were reported for the staurosporine-like compound Z-705, for which *S. cerevisiae* strains expressing chimeric yeast-filamentous fungal PKC of *Magnaporthe grisea* and *A. nidulans* were constructed. Here Z-705 decreased Pmk1/MpkA phosphorylation for *M. grisea* and *A. nidulans*, but not in the wild type *S. cerevisiae*, suggesting Z-705 to be a filamentous fungi specific PKC inhibitor which directly affects the CWI pathway [81]. Still, no *in vivo* data is available to evaluate off-target binding of this compound, which is a noteworthy consideration for further drug discovery efforts due to the known toxicity of the kinase inhibitors. In this study, we did not evaluate fungal specific kinase inhibitors, which complicates drug screening efforts and efficacy testing. Most reported kinase inhibitors do affect both mammalian models, as well as invertebrate models such as *G. mellonella*. For example, pre-treatment with the JNK inhibitor SP600125 delayed and reduced the immune response in *G. mellonella* larvae [82]. Therefore, more fungal specific analogues of this parent molecule should be rationally designed using structure-activity relationships (SAR) strategies to circumvent non-specific binding of the molecules to undesirable proteins such as other human kinases. Other interesting scaffolds could also be identified in the future when exploring novel techniques, such as pH-dependent protein precipitation, to study ligand-protein interactomes and identify potential novel target proteins [83,84]. The novel analogues should then again be tested for their *in vitro* activity against *M. mycetomatis* and their *in vivo* efficacy in the invertebrate and mouse models for eumycetoma, before they can finally be evaluated in clinical trials. Altogether, this study is the first step in exploring kinase inhibitors as potential drug candidates for the drug discovery pipeline for mycetoma.

To conclude, our bioinformatical approach identified 132 predicted kinases in the *M. mycetomatis* kinome, of which 4 kinases were predicted as essential and not present in the human kinome. Among these, pek1 (MMYC01_208733) and mhk1 (MMYC01_207542), components of the CWI pathway were identified and suggested as promising target. Drug screening of kinase inhibitors revealed five compounds able to inhibit fungal growth by targeting MAP kinases, including parts of the CWI pathway, and confirm kinase inhibitors as promising compounds to study further within the Open Source MycetOS project.

## Supporting information

S1 Data Molecular Docking of MMYC01_208733 (Pek1/Mkk2) and MMYC01_207542 (mkh1) of *Madurella mycetomatis.* (ZIP)

S1 Table Kinannote classification of predicted *M. mycetomatis* kinases into groups and families. (XLSX)

S2 Table The overview of the manually curated Kinannote kinome prediction of *M. mycetomatis*, kinase classification and orthologue identification compared to *A. nidulans* and *N. crassa.* (XLSX)

S3 Table Venn diagram depicting the clusters of orthologues in *M. mycetomatis* and *H. sapiens* based on OrthoVenn3 analysis. (XLSX)

S4 Table Kinases GO analysis. (XLSX)

S5 Table Candidate drug target list. Kinases were divided in four categories based on predicted orthologues with the human kinome and whether the kinases were predicted as essential for fungal viability. For each gene the respective raw read counts of the transcriptomic profiling are provided per time point per sample.
(XLSX)

S6 Table RNA-seq Total tag counts per sequence library. (XLSX)

S7 Table Mapping QC of RNA-seq reads per sequence library. (XLSX)

S8 Table Data summary of the KCGS2.0 plate containing kinase data and coverage of the respective compounds as provided by suppliers’ documentation, indicated in red [21]. The data summary was supplemented with growth percentages of *M. mycetomatis* upon exposure to the respective compounds.
(XLSX)

S9 Table Data summary of the MycetOS compounds. (XLSX)

S1 Fig Gene Ontology enrichment of all identified kinases. (TIF)

S2 Fig Hierarchical clustering of the expression of all kinases genes *in vitro.* Data source provided as DataSource_SupplFig_2.
(JPG)

S3 Fig Hierarchical clustering of the expression of all *M. mycetomatis* kinases genes during infection in *G. mellonella* larvae. The data source provided as DataSource_SupplFig_3.
(JPG)

S4 Fig 3D and 2D protein-ligand interaction of MAP_kinase_mkh1 with tyrphostin A9 and GW434756X, and MAP_kinase_skh1 with tyrphostin A9 and GW434756X. (PNG)
