# Supplementary material for: Kinome analysis of Madurella mycetomatis identified kinases in the cell wall integrity pathway as novel potential therapeutic drug targets in eumycetoma caused by Madurella mycetomatis
Source: PLoS Negl Trop Dis. 2025 Sep 4;19(9):e0013482. doi: 10.1371/journal.pntd.0013482 (PMC12425257; doi:10.1371/journal.pntd.0013482)
Supplement: S1 Data — (ZIP) [file pntd.0013482.s001.zip › S1_Data.docx]

**Supplemental data 1:** **Molecular Docking of MMYC01_208733 (Pek1/Mkk2) and MMYC01_207542 (mkh1) of Madurella mycetomatis**

**Methodology:**

**Protein structure prediction:**

The homology modeling of the proteins MMYC01_208733 (Pek1/Mkk2) and MMYC01_207542 (mkh1) of Madurella mycetomatis, was carried out using the most advanced AI-generated DeepMind AlphaFold**^1^** (<https://alphafoldserver.com/>) due to the unavailability of experimental models. This cutting-edge technology utilizes deep learning algorithms to predict the three-dimensional structure of proteins based on their amino acid sequences. The ERRAT**^2^** and PROCHECK**^3^** Ramachandran plot analyses were performed to assess the quality and stereochemical validation of the protein structure. ERRAT, a structure validation tool, evaluates the overall quality of the model by analyzing non-bonded atomic interactions, generating an error function-based quality score. Values above 90% indicate a reliable structure. The protein model in PDB format was uploaded to the ERRAT server for evaluation. PROCHECK was used to analyze the backbone dihedral angles (ϕ and ψ) through the Ramachandran plot, assessing stereochemical quality by categorizing residues into favored, allowed, and disallowed regions. The model was uploaded to the PROCHECK server, and the plot was generated to verify conformational integrity, ensuring the structure adhered to standard stereochemical parameters. These analyses provided critical insights into the reliability and accuracy of the modeled protein structure.

**Molecular docking:**

The target protein refinement was performed using CHIMERA V1.16**^4^** to ensure high-quality input for the docking study. Standard residues present in the protein were minimized using the AMBER force field, while non-standard residues were minimized using AM1-BCC semi-empirical charges to maintain proper electrostatic interactions. Non-essential residues, such as water, co-crystal ligands, and unnecessary chains, were removed, as they may interfere with the docking procedure. The structures of the ligands were drawn in Marvin Sketch (<http://www.chemaxon.com/>), and hydrogens were added. Optimal 2D and 3D clean configurations were selected, and possible conformers were generated from which the lowest-energy conformer was chosen and saved in MOL2 format. Then, the structures were optimized using CHIMERA with the AM1-BCC force field. Proteins and ligands were converted to PDBQT format using AutoDockTools**^5^**, which can be read by the Vina tool. In AutoDockTools, the rotatable bonds in the ligands were identified and made flexible during the docking study for conformational variability. The protein was kept rigid, while the ligand's flexibility was explicitly considered through the use of flexible rotatable bonds. It is important to note that docking was performed without explicit solvent conditions. Active site amino acids were identified using the CASTp server**^6^**. Then the Grid box was defined around the active site, and the grid dimensions were optimized to encompass the protein-ligand interactions. The docking protocol was meticulously designed to reflect insights into interactions between protein-ligand complexes. We used Autodock Vina V. 1.2.6**^7,8^** for molecular docking. The docking parameters were optimized by multiple docking runs using various grid sizes and based on different tools to identify the best binding pose and replicable results like spacing (0.375 Å), num_modes (9), energy range (3), exhaustiveness (16), and other default configurations. Docking results were visualized through Biovia Discovery Studio visualization tool **(**<https://www.3ds.com/products-services/discovery-studio/>) and Maestro 12.3 (Academic Edition) (<https://www.schrodinger.com/maestro>) for structural analysis, and binding interactions were noted in the table.

**Result:**

**Protein structure prediction:**


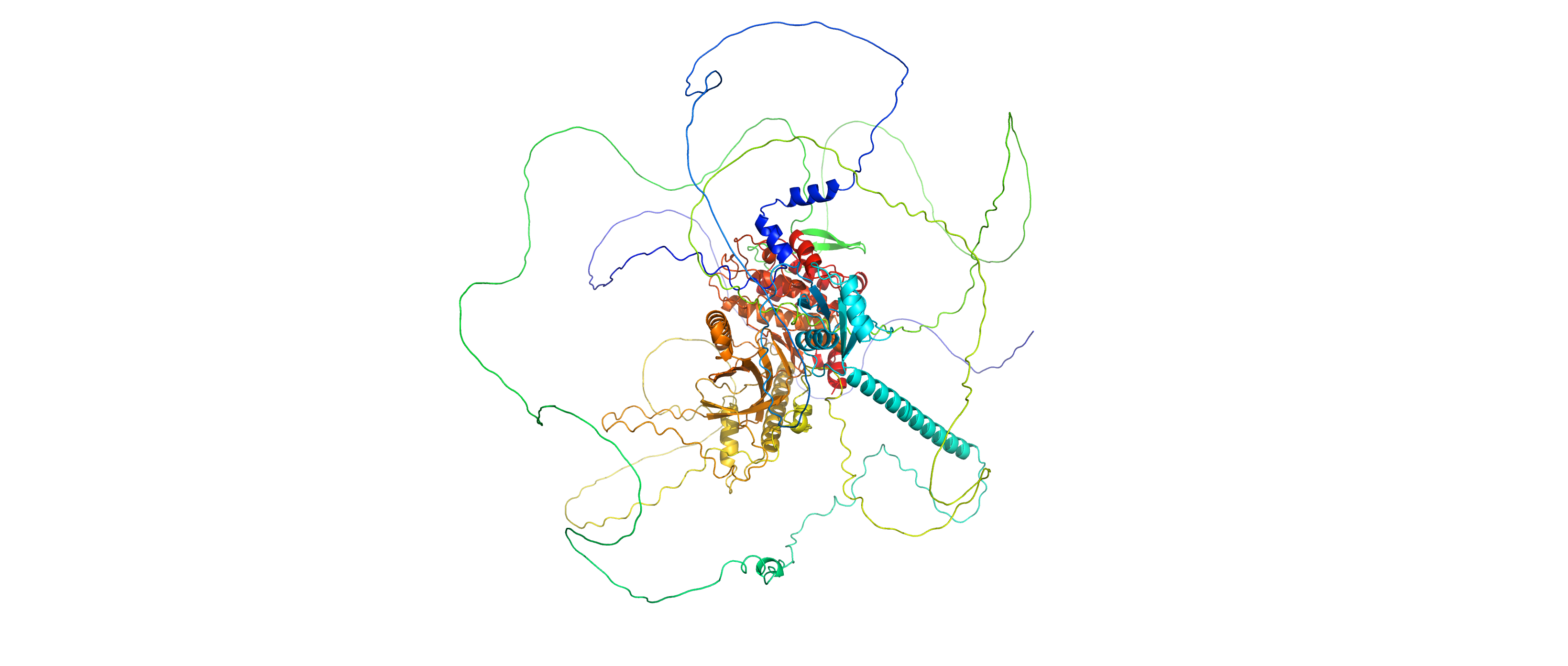
**
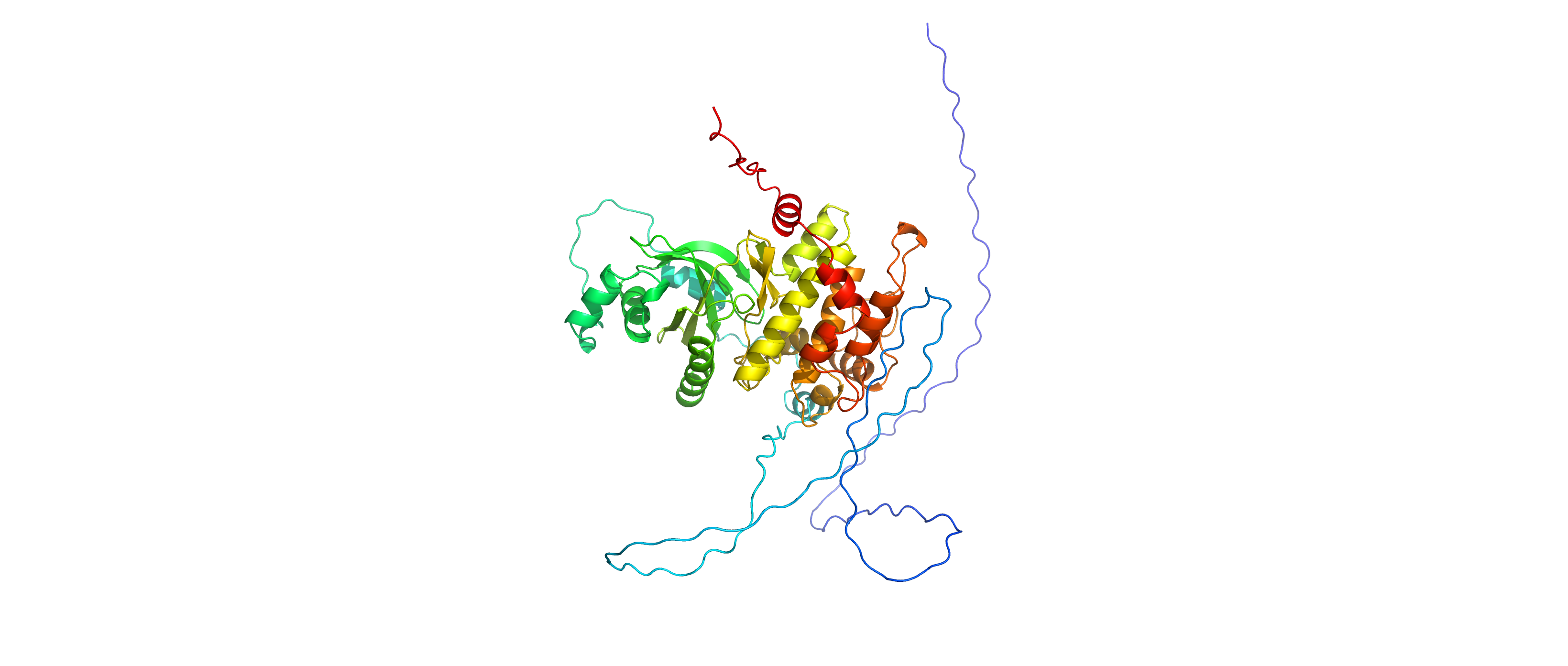
**

**A** **B**

Figure 1: Structure of Modelled protein of MAP kinase mkh1 **(A)** and MAP kinase skh1/pek1 **(B)**

The ERRAT plot shown in the image (figure 2) represents the quality assessment of a MAP Kinase models generated using AlphaFold. ERRAT is a structure validation tool that assesses the overall quality of a protein model by calculating the error values at different residue positions, highlighting regions with potentially unreliable structures. In the presented plots, the x-axis represents the residue number (window center), while the y-axis denotes the error value.

**A B**

**Figure 2:** ERRATE plot of Modelled protein of MAP kinase mkh1 **(A)** and MAP kinase skh1/pek1 **(B)**

The plot contains multiple segments, suggesting that the MAP Kinase model is extensive and comprises several domains. The red bars indicate regions with extremely high error values (>99%), signifying problematic areas that may require structural refinement. These regions likely correspond to loop regions, flexible termini, or incorrectly modeled secondary structures. The yellow bars indicate residues with moderately high error values (>95%), suggesting less severe but still notable structural inaccuracies. ERRAT operates by analyzing the distribution of nonbonded atom pairs and comparing the observed patterns against established high-resolution crystallographic structures. It primarily focuses on the spatial arrangement of atoms to detect regions of high error, thereby providing insight into potential structural inaccuracies. The black and grey bars represent lower error values, with most of the residues falling within the acceptable range, indicating that a significant portion of the model is structurally reliable. The presence of multiple peaks in the plot suggests that some areas of the protein might be poorly modeled due to insufficient template availability in AlphaFold’s training dataset or inherent flexibility in the kinase domain. Two critical confidence thresholds, set at 95% and 99%, demarcate regions where error values exceed acceptable limits. Residues with error values above these thresholds are considered structurally unreliable and warrant further investigation. These high-error regions often correspond to areas of steric clashes, improper hydrogen bonding, incorrect side-chain orientations, or unfavorable backbone conformations. The overall quality factor provided by ERRAT is a key metric in structural validation, expressed as the percentage of the protein for which the error values fall below the 95% rejection limit. This metric serves as an indicator of the overall reliability of the model, with values above 95% typically associated with high-resolution experimental structures. For protein models determined at resolutions between 2.5 to 3.0 Å, an average quality factor around 91% is considered acceptable. ERRATE plot of the Modelled protein of MAP kinase MKH1 shows high-error regions around residues 20-40, 140-160, 460-480, and 740-780, corresponding to critical flexible or disordered segments, possibly within activation loops or linker regions, which are known to exhibit high variability in kinases. Similarly, the C-terminal region (940-1080) also exhibits elevated error values, possibly indicating the presence of unstructured or functionally flexible domains. The reliable regions of the model, characterized by low error values, likely correspond to conserved structural elements such as the catalytic core of the kinase. The ERRAT plot of MAP kinase skh1/pek1 exhibits an overall quality factor of 91.369, suggesting that a moderately high-quality structural model with minor deviations. Although this value aligns well with the acceptable range for medium-resolution structures, regions exceeding the error thresholds should be scrutinized for possible refinements.


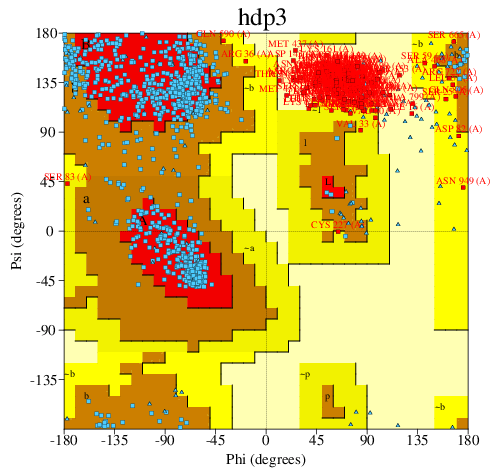

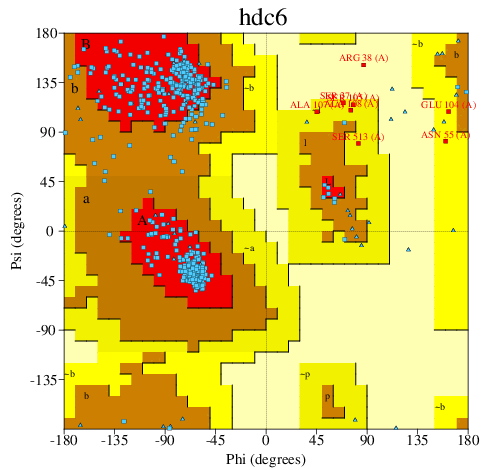


**A B**

**Figure 3.** Ramachandran plot analysis of the modelled protein of MAP kinase mkh1 **(A)** and MAP kinase skh1/pek1 **(B)**, displaying the distribution of phi (Φ) and psi (Ψ) dihedral angles for non-glycine and non-proline residues. Blue dots represent individual residues, while labeled residues (red text) indicate those outside favored regions. This plot provides insight into the overall structural quality and backbone conformation of the modeled protein.

The Ramachandran plot for the modeled MAP kinase mkh1 and MAP kinase skh1/pek1 provides critical insights into the stereochemical quality and conformational stability of the structure. The analysis indicates that out of a total of 1,253 residues of MAP kinase mkh1, 661 residues (83.4%) fall within the most favored regions, representing the core allowed phi (Φ) and psi (Ψ) dihedral angles where steric hindrance is minimal, thus confirming a well-refined and reliable model. Additionally, 205 residues (9.7%) are positioned in the additional allowed regions, suggesting some flexibility in secondary structure elements, while 41 residues (3.9%) occupy the generously allowed regions, indicating minor deviations that are still permissible within the Ramachandran constraints. A small fraction, 136 residues (3.0%), are found in the disallowed regions, which may correspond to regions of structural strain, errors, or functionally significant conformations such as active site residues or loop regions undergoing dynamic movements. The presence of 120 glycine residues, which inherently possess greater conformational flexibility due to the absence of a side chain, explains some deviations observed in the plot. Likewise, 88 proline residues contribute to rigid structural constraints, typically found in turns and loops. The presence of only two end residues (excluding glycine and proline) further validates the compact nature of the protein model. The overall statistics indicate that the MAP kinase mkh1 model has an acceptable stereochemical quality, with a majority of residues residing in favored regions and only a minor fraction in disallowed conformations.

For the modelled protein MAP kinase skh1/pek1, 87.2% of residues fall within the most favored regions, slightly below the expected >90% for a high-quality model, with 10.9% in allowed regions and only 0.2% (one residue) in disallowed regions, indicating a largely stable structure with minor deviations. G-factor analysis shows an overall score of -0.08, indicating reasonable conformational integrity, though the omega angle (-0.93) is slightly unusual, possibly affecting certain regions. To evaluate the quality of AP kinase kinase Skh1/Pek1, similar Ramachandran plot statistics and G-factor analyses are required, ensuring that over 90% of residues fall in favored regions and that disallowed regions are minimal (<1%). If STESTE7MKK exhibits a distribution similar to or better than hdc6, with stable G-factors and minimal outliers, it would be considered a well-modeled structure

**Molecular Docking**

Molecular docking-based virtual screening was carried out for selected compounds against MAP Kinase Skh1 and Mkh1 using AutoDock Vina version 1.2.6. Binding site residues identified from Castp server in the active site of MAP Kinase Skh1/Pek1 are MET290A, LEU219A, GLY220A, GLU221A, GLY222A, ALA223A, GLY224A, GLY225A, VAL227A, ALA240A, LYS242A, ILE244A, THR245A, THR246A, ILE256A, GLU291A, PHE292A, CYS293A, GLU294A, GLY296A, SER297A, ASP299A, LYS303A, SER300A, ASP340A, LYS342A, PRO343A, SER344A, ASN345A, LEU347A, ASP358A, VAL361A, SER362A, PHE365A, THR367A, ASN372A, THR373A, PHE374A, GLY376A, THR377A, TYR379A, TYR380A, GLU406A, PHE412A While in MAP Kinase Mkh1 are ASP642A, ARP753A, ALA754A, ARG756A, ALE969A, ALY970A, AYS971A, ALY972A, AHR973A, AAL977A, ALA990A, AYS992A, AAL1031A, AEU1047A, ALU1048A, ALE1050A, AER1054A, AER1057A, AYS1095A, ASP1097A, ASN1098A, AEU1100A, AER1110A, ASP1111A. According to the active site, grid centers were represented around the native ligand, which were found for skh1, were X= -7.1348, Y= 2.2996, Z= -5.4004, for mkh1 are X= -3.305, Y= -3.2464, Z= -0.1855, and the grid size was set to X=Y=Z=25 for all proteins. The docking results were analyzed in terms of binding energy, affinity, and the type of interactions at the active site of the selected targets. The results were represented in the table 1 and 2.

| Sr. No | Name of Complex | Binding energy (kcal/mol) | Type of interaction: Residue ID (Distance) |
| --- | --- | --- | --- |
| 1 | GW832467X | -11.419 | Hydrophobic Interactions: VAL977A (3.47), ASP1097A (3.53), LEU1100A (3.76) |
|  |  |  | Hydrogen Bonds: SER640A (2.62), SER640A (2.27), SER1054A (3.03), SER1054A (3.3), ASP1097A (2.45), ASP1111A (3.09) |
|  |  |  | π-Cation Interactions: ARG1060A (4.06) |
| 2 | STAUROSPORINE | -6.11 | Hydrophobic Interactions: ASP642A (4), ALA646A (3.62), THR973A (3.37), ILE1114A (3.47) |
|  |  |  | Hydrogen Bonds: SER93A (2.35), ASP642A (3.1), LYS1095A (3.29) |
| 3 | TYRPHOSTIN_A9 | -6.989 | Hydrophobic Interactions: TRP753A (3.67) |
|  |  |  | Hydrogen Bonds: SER1054A (2.07), ARG1060A (2.24), ASP1097A (2.05), ASP1111A (3.06) |
| 4 | GW434756X | -8.072 | Hydrophobic Interactions: VAL977A (3.36), ASP1097A (3.77), LEU1100A (3.23) |
|  |  |  | Hydrogen Bonds: ARG756A (2.77), ARG756A (3.16), ASP1111A (2.87), ASP1111A (2.67) |
|  |  |  | π-Cation Interactions: ARG756A (4.5), ARG756A (3.93) |

**Table No. 1: Protein-ligand interaction of MAP Kinase Mkh1**

| Sr. No | Name of Complex | Binding energy (kcal/mol) | Type of interaction: Residue ID(Distance) |
| --- | --- | --- | --- |
| 1 | GW709042A | -11.528 | Hydrophobic Interactions: LEU219A (3.77), LEU219A (3.81), ALA223A (3.69), LEU347A (3.44), PHE412A (3.99), PHE412A (3.49) |
|  |  |  | Hydrogen Bonds: SER297A (2.7), SER344A (2.8) |
|  |  |  | p-Stacking: TYR379A (4.86) |
|  |  |  | p-Cation Interactions: LYS342A (3.93) |
|  |  |  | Halogen Bonds: SER300A (3.79) |
| 2 | STAUROSPORINE | -7539 | Hydrophobic Interactions: THR373A (3.99) |
|  |  |  | Hydrogen Bonds: ALA223A (2.72), GLY224A (3.35), GLY224A (2.93), ASP299A (3.21) |
|  |  |  | π-Cation Interactions: LYS342A (3.5) |
| 3 | TYRPHOSTIN_A9 | -7.049 | Hydrophobic Interactions: LEU219A (3.55), VAL227A (3.55) |
|  |  |  | Hydrogen Bonds: SER300A (2.67), SER344A (2.55), ASP358A (3.08) |
| 4 | GW434756X | -8.052 | Hydrophobic Interactions: ILE244A (3.95), PHE365A (3.82), THR367A (3.54), THR377A (3.62) |
|  |  |  | Hydrogen Bonds: LYS242A (2.61), ASP370A (3.08) |

**Table No. 2: Protein-ligand interaction of MAP Kinase Skh1**

Figure 4 illustrates the structures of MAP kinase Mkh1 and Skh1, along with representative compounds that display both 3D and 2D structures, showing specific detailed interactions such as hydrogen bonding, π-stacking, salt bridges, and hydrophobic interactions.

**
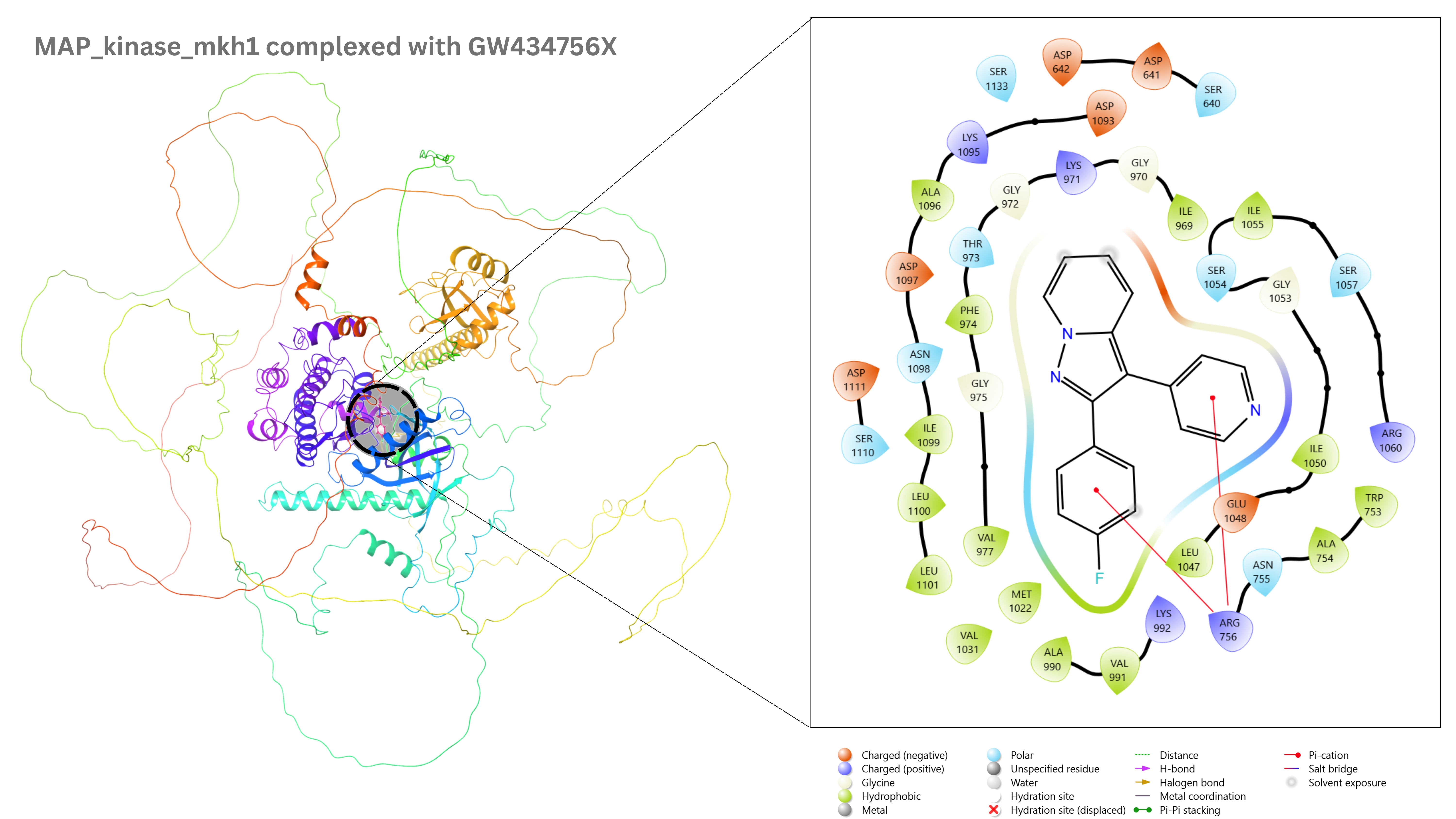
**

**A**

**
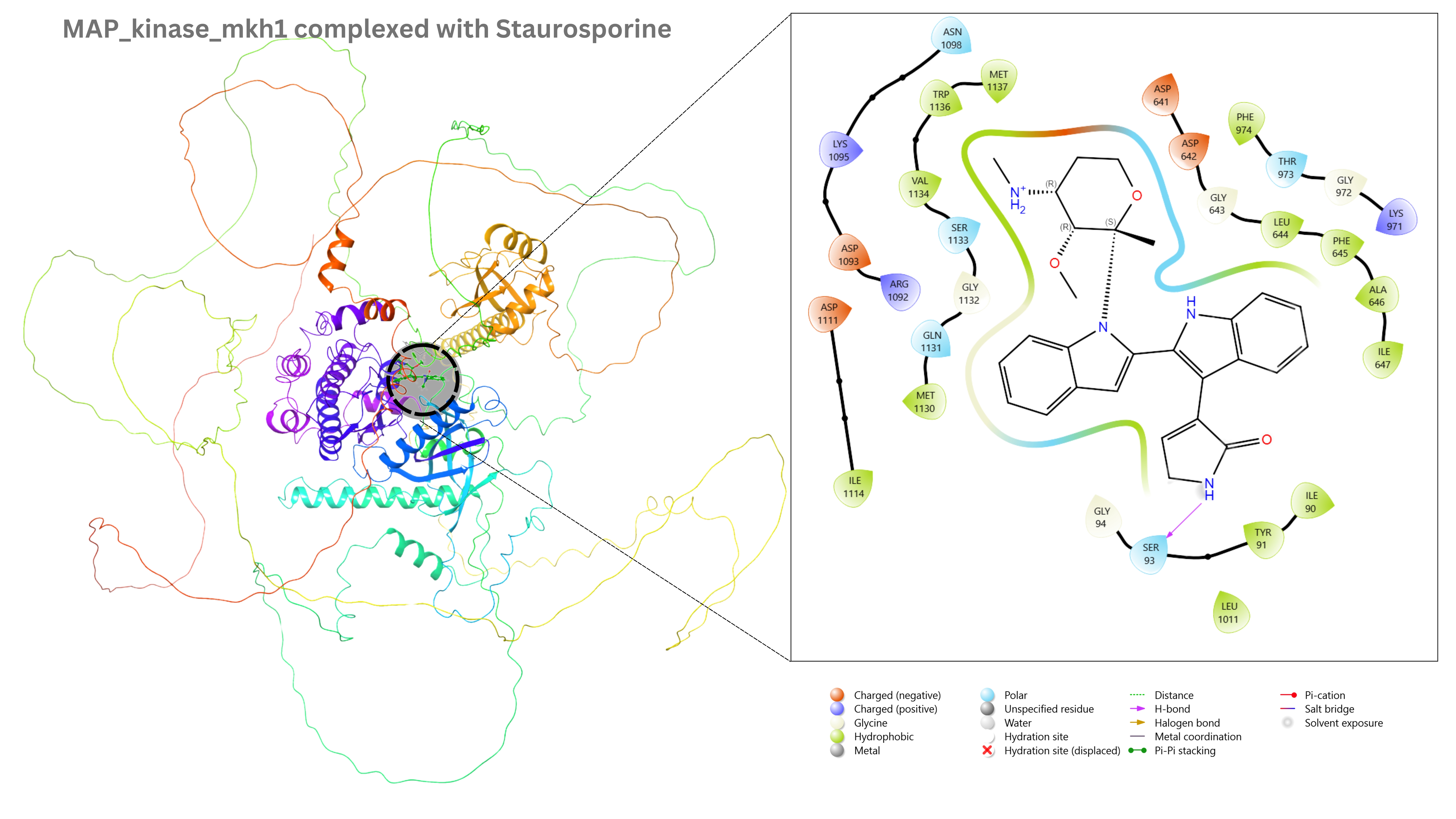
**

**B**

**
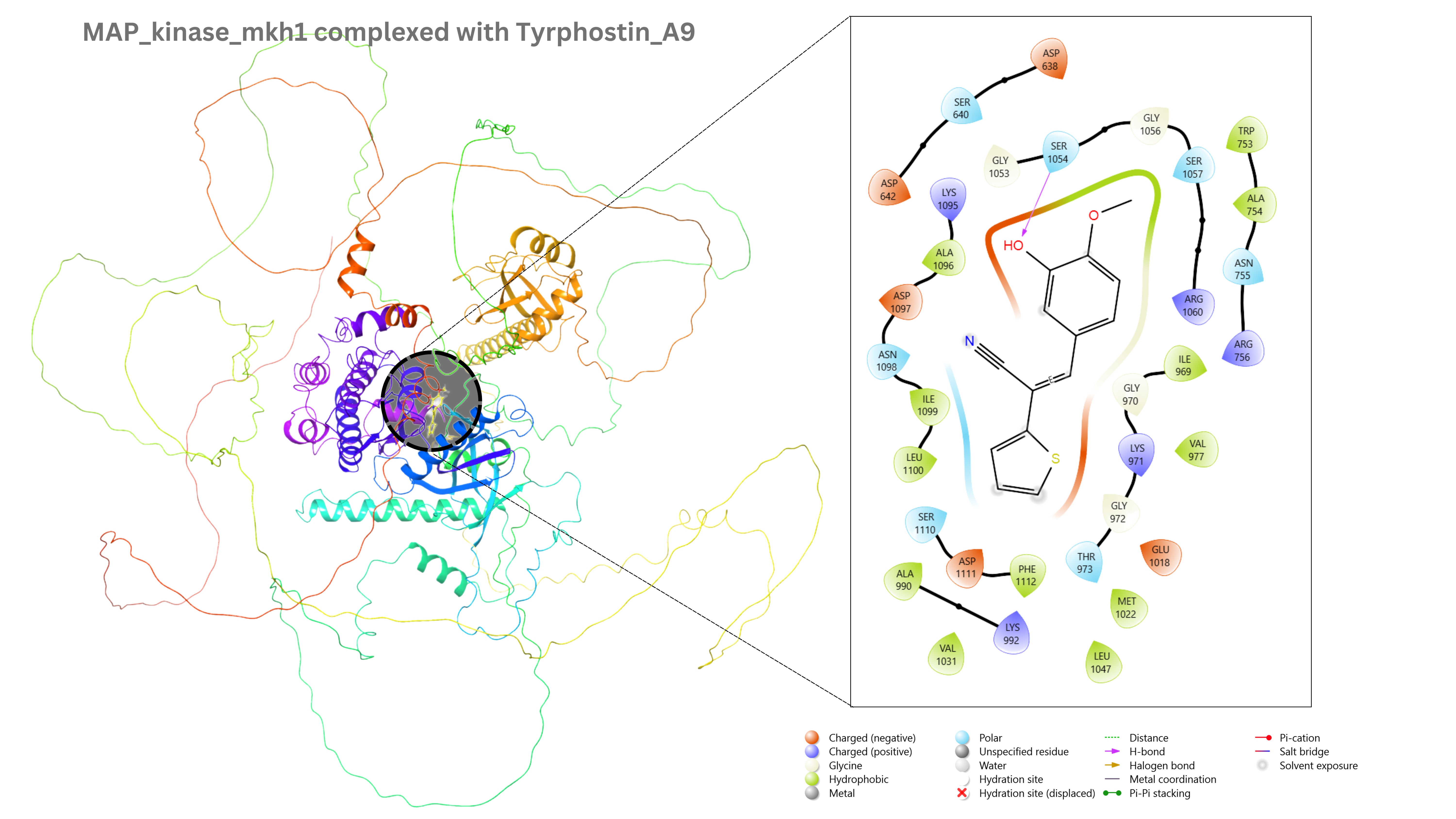
**

**C**

**
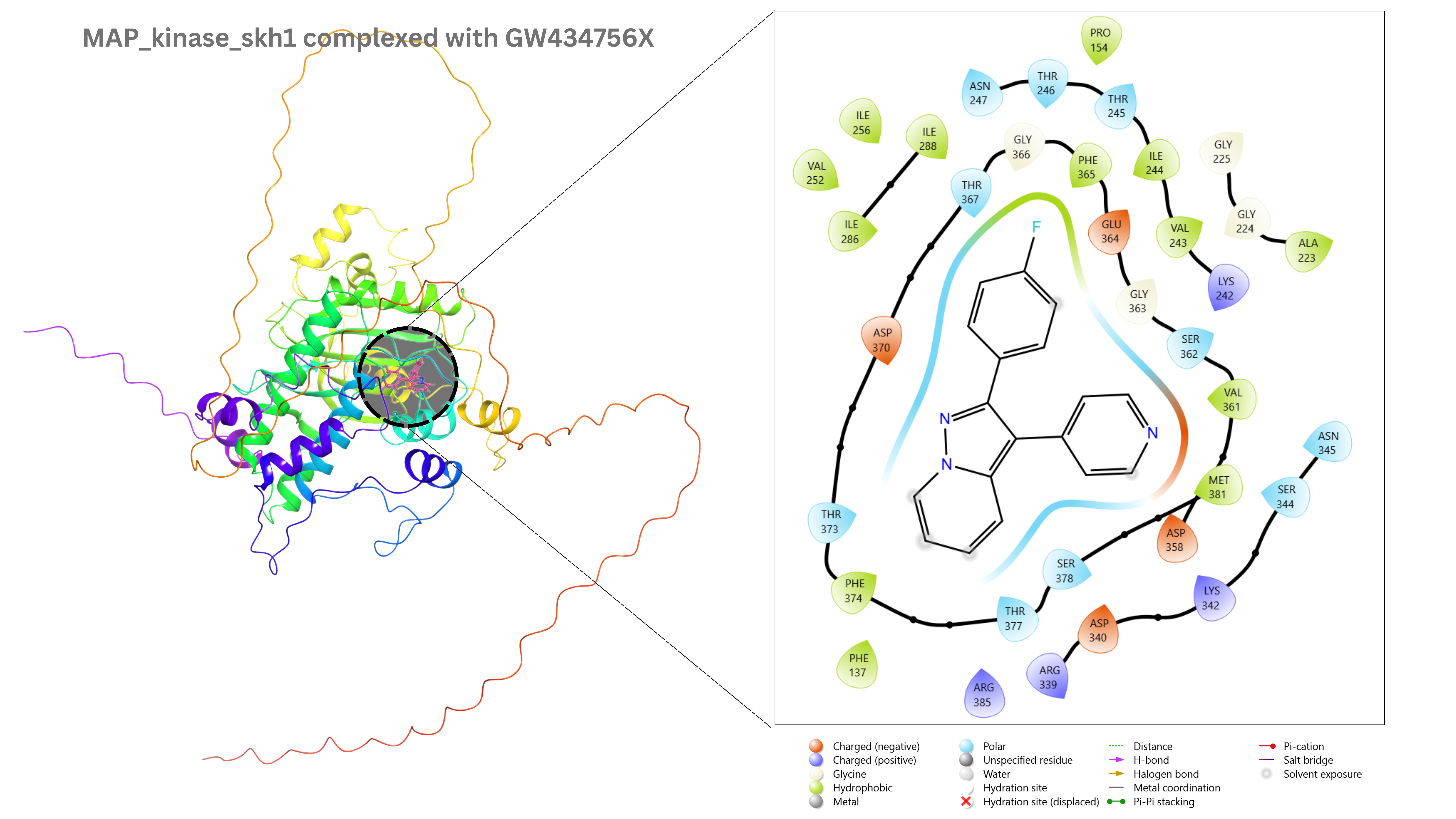
**

**D**

**
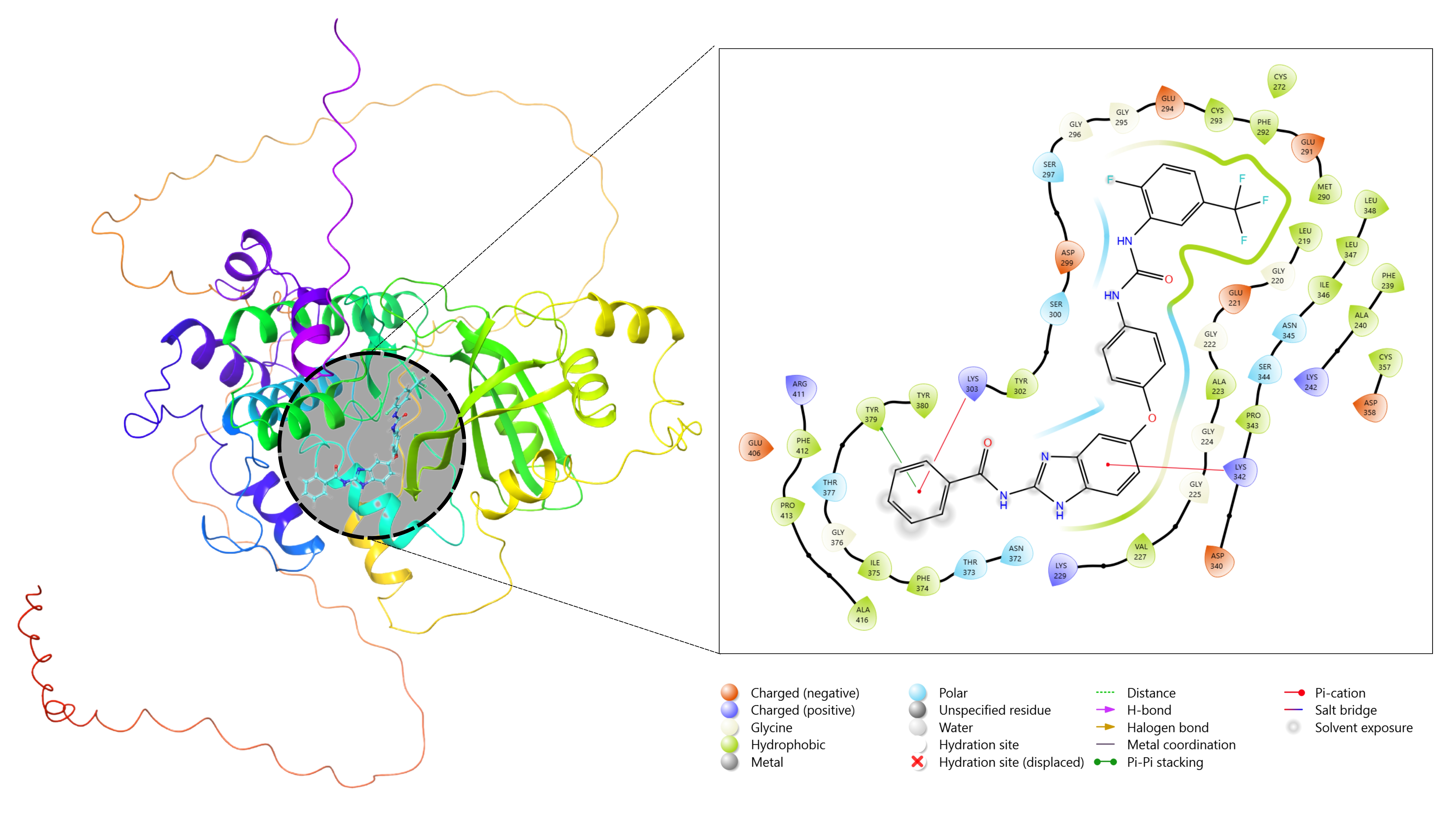
**

**E**

**
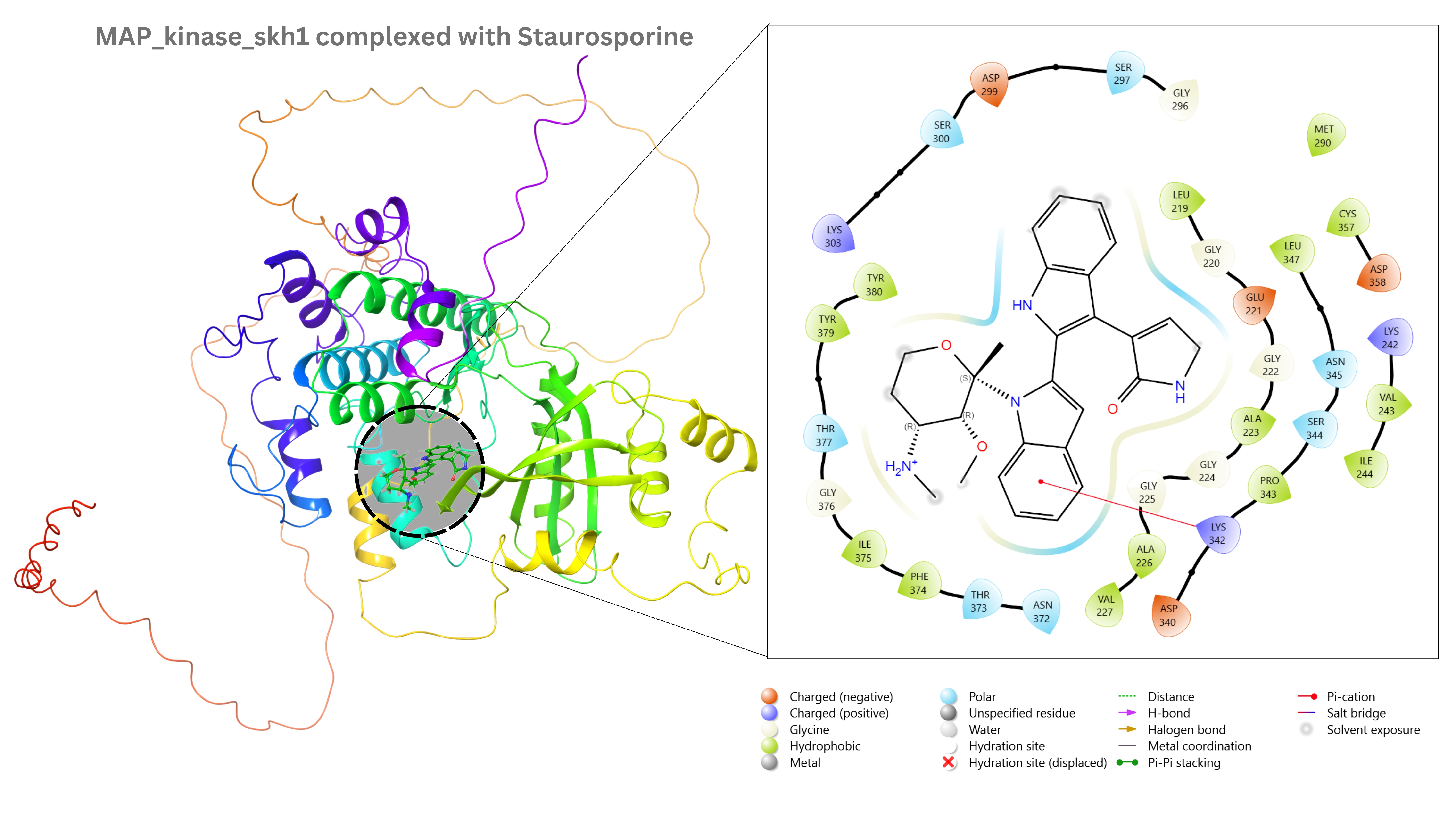
**

**F**

**
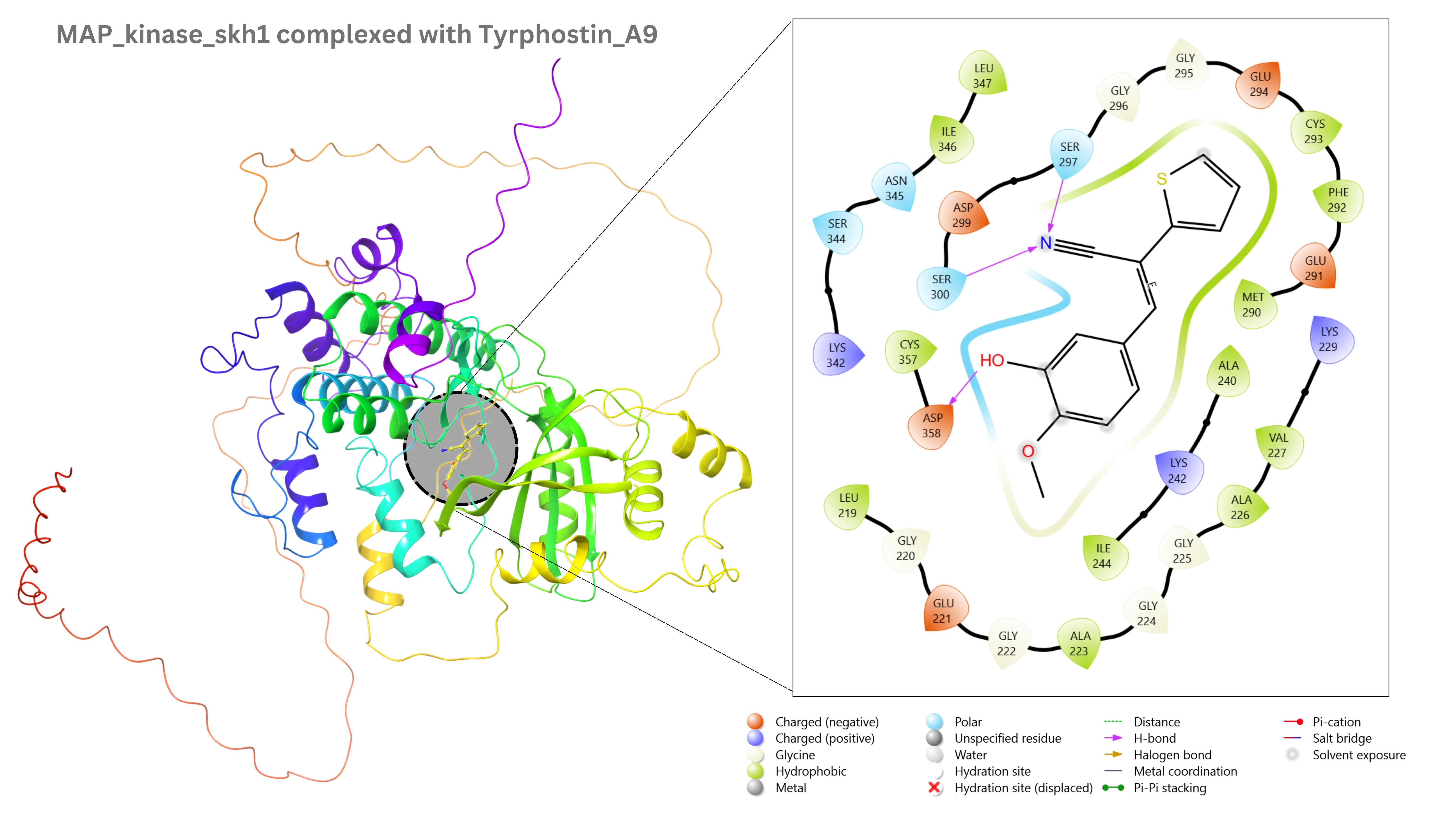
**

**G**

**
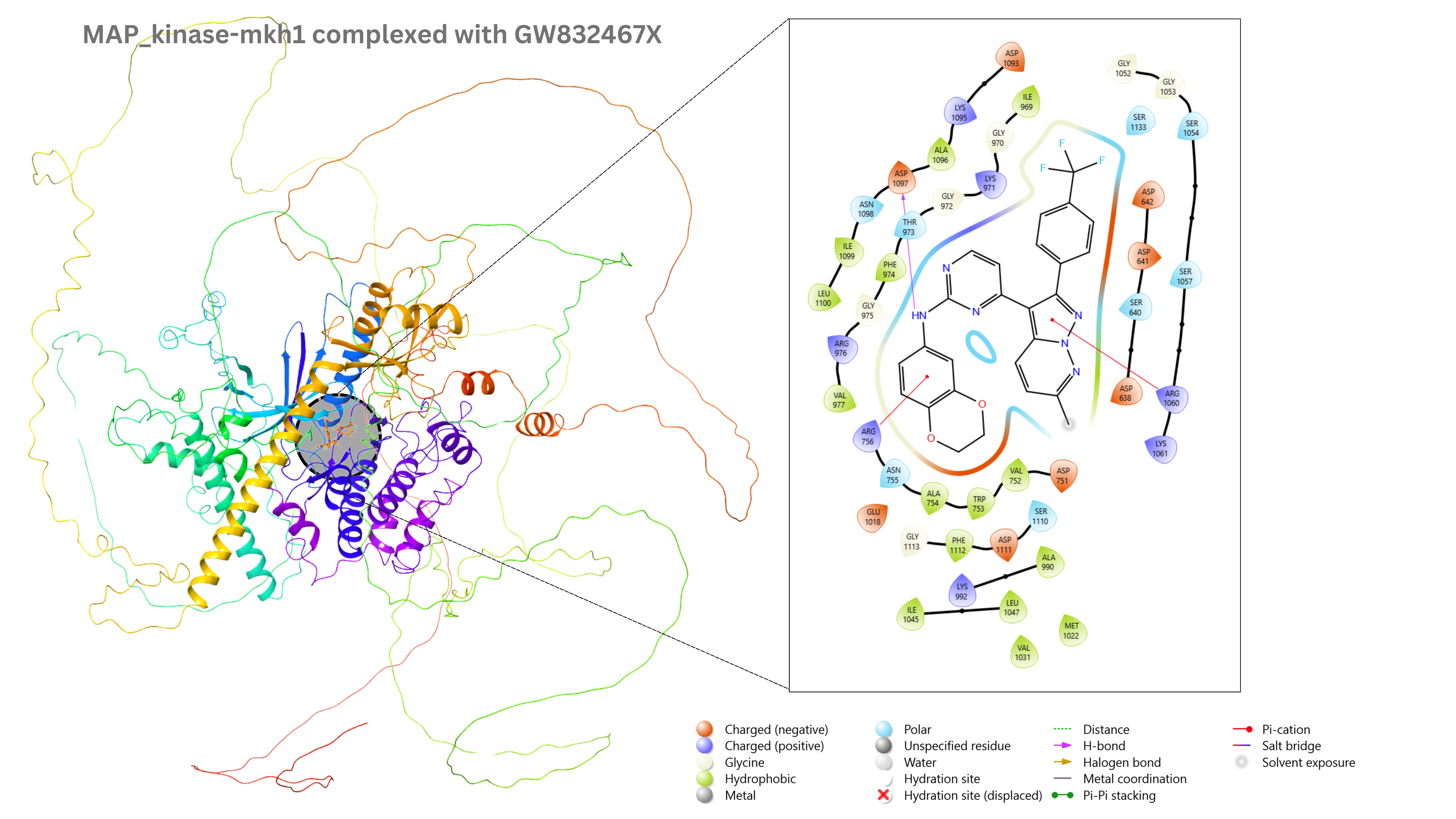
**

**H**

**Figure 4:** 3D and 2D protein-ligand interaction of MAP_kinase_mkh1 with GW434756X (A), MAP_kinase_mkh1 with Staurosporine (B), MAP_kinase_mkh1 with Tyrphostin_A9 (C), MAP_kinase_skh1 with GW434756X (D), MAP_kinase_skh1 with GW709042A (E), MAP_kinase_skh1 with Staurosporine (F), MAP_kinase_skh1 with Tyrphostin_A9 (G), and MAP_kinase-mkh1 with GW832467X (H).

**References:**

1. Abramson, J., Adler, J., Dunger, J. *et al.* Accurate structure prediction of biomolecular interactions with AlphaFold 3. *Nature* **630**, 493–500 (2024). https://doi.org/10.1038/s41586-024-07487-w

2. Colovos C, Yeates TO. Verification of protein structures: patterns of nonbonded atomic interactions. Protein Sci. 1993 Sep;2(9):1511-9. doi: 10.1002/pro.5560020916. PMID: 8401235; PMCID: PMC2142462.

3. Laskowski R A, MacArthur M W, Moss D S, Thornton J M (1993). PROCHECK - a program to check the stereochemical quality of protein structures. *J. App. Cryst.*, **26**, 283-291.

4. Pettersen EF, Goddard TD, Huang CC, Couch GS, Greenblatt DM, Meng EC, Ferrin TE. UCSF Chimera--a visualization system for exploratory research and analysis. J Comput Chem. 2004 Oct;25(13):1605-12. doi: 10.1002/jcc.20084. PMID: 15264254.

5. Morris, G. M., Huey, R., Lindstrom, W., Sanner, M. F., Belew, R. K., Goodsell, D. S. and Olson, A. J. (2009) [Autodock4 and AutoDockTools4: automated docking with selective receptor flexiblity.](http://www.ncbi.nlm.nih.gov/pmc/articles/PMC2760638/) J. Computational Chemistry 2009, **16**: 2785-91.

6. Wei Tian, Chang Chen, Xue Lei, Jieling Zhao, Jie Liang, CASTp 3.0: computed atlas of surface topography of proteins, Nucleic Acids Research, Volume 46, Issue W1, 2 July 2018, Pages W363–W367, <https://doi.org/10.1093/nar/gky473>

7. Jerome Eberhardt, Diogo Santos-Martins, Andreas F. Tillack, and Stefano Forli *Journal of Chemical Information and Modeling* **2021** *61* (8), 3891-3898 DOI: 10.1021/acs.jcim.1c00203

8. Gabriella O Estevam, Edmond Linossi, Jingyou Rao, Christian B Macdonald, Ashraya Ravikumar, Karson M Chrispens, John A Capra, Willow Coyote-Maestas, Harold Pimentel, Eric A Collisson, Natalia Jura, James S Fraser, Mapping kinase domain resistance mechanisms for the MET receptor tyrosine kinase via deep mutational scanning, eLife, 10.7554/eLife.101882.3, **13**, (2025).
