# Supplementary figures and images for: Kinome analysis of Madurella mycetomatis identified kinases in the cell wall integrity pathway as novel potential therapeutic drug targets in eumycetoma caused by Madurella mycetomatis
Source: PLoS Negl Trop Dis. 2025 Sep 4;19(9):e0013482. doi: 10.1371/journal.pntd.0013482 (PMC12425257; doi:10.1371/journal.pntd.0013482)

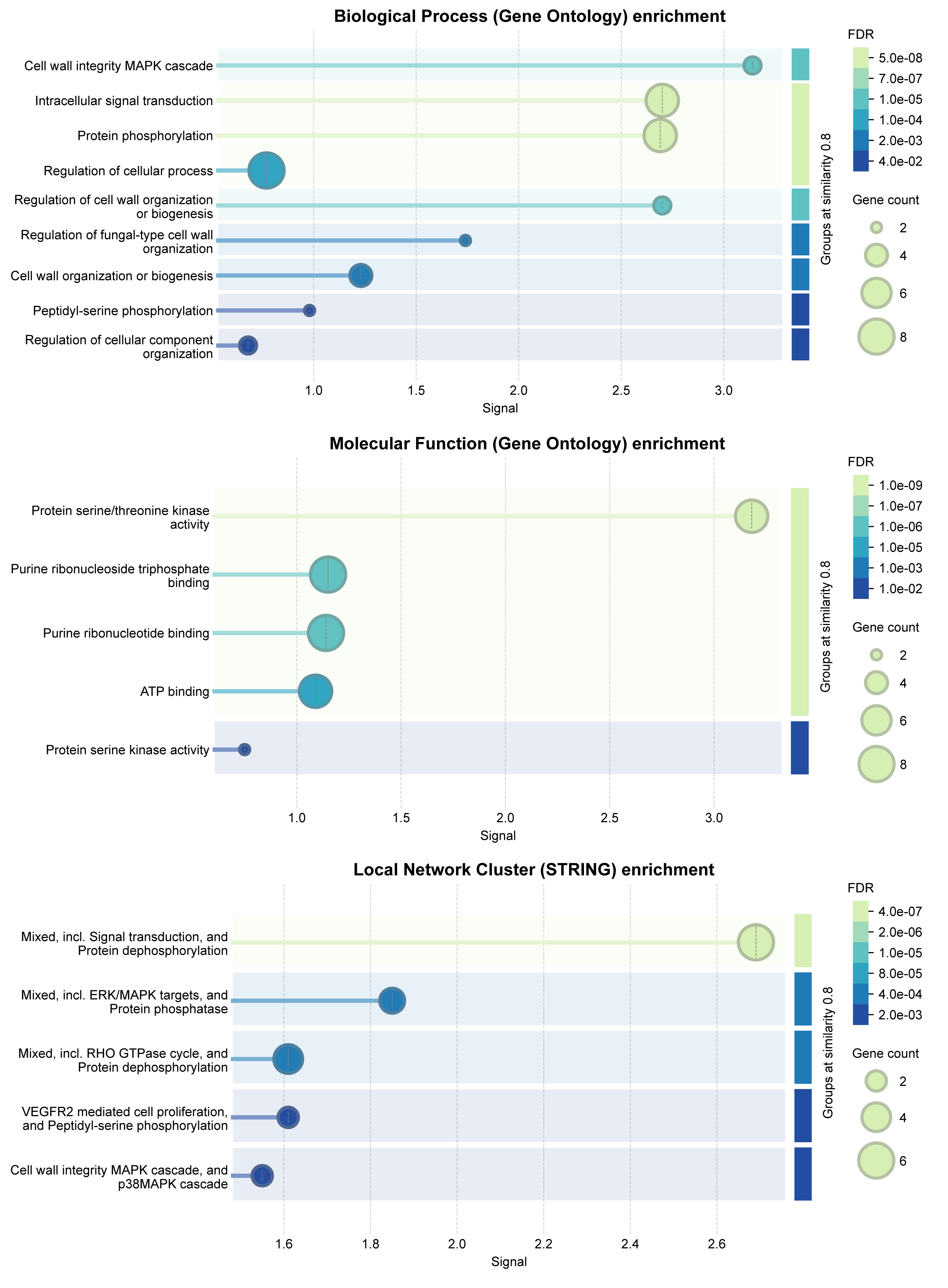

Supplement: S1 Fig — (TIF) [file pntd.0013482.s011.tif]

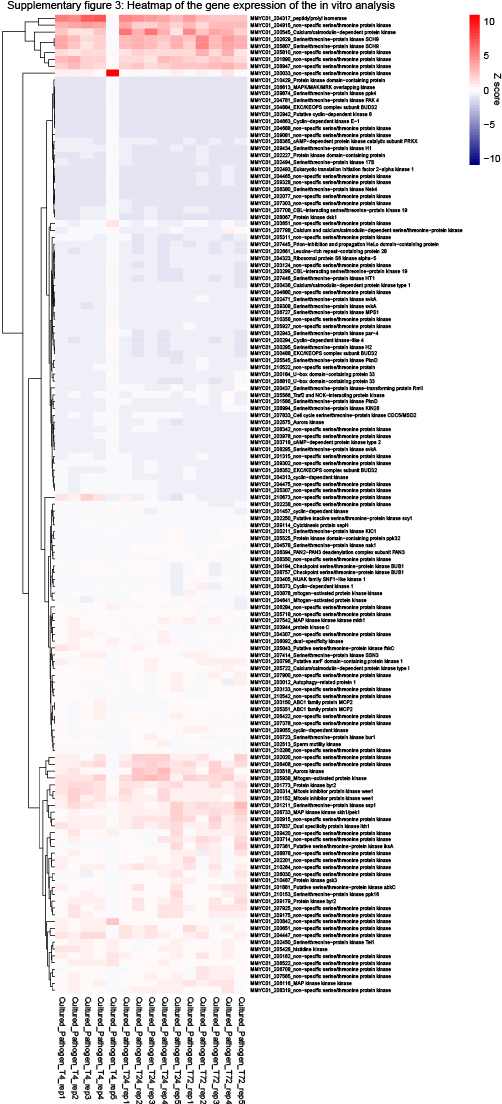

Supplement: S2 Fig — Data source provided as DataSource_SupplFig_2. (JPG) [file pntd.0013482.s012.jpg]

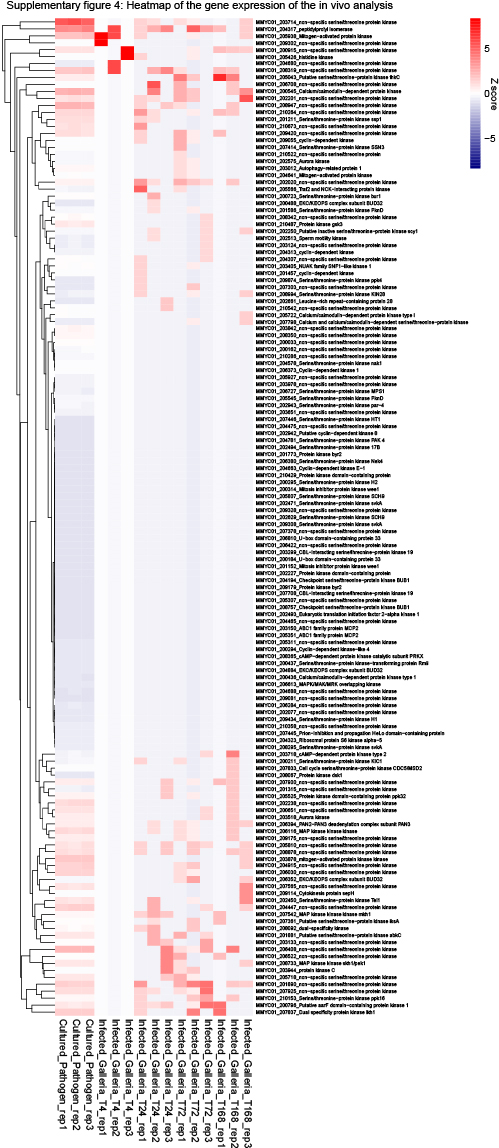

Supplement: S3 Fig — The data source provided as DataSource_SupplFig_3. (JPG) [file pntd.0013482.s013.jpg]

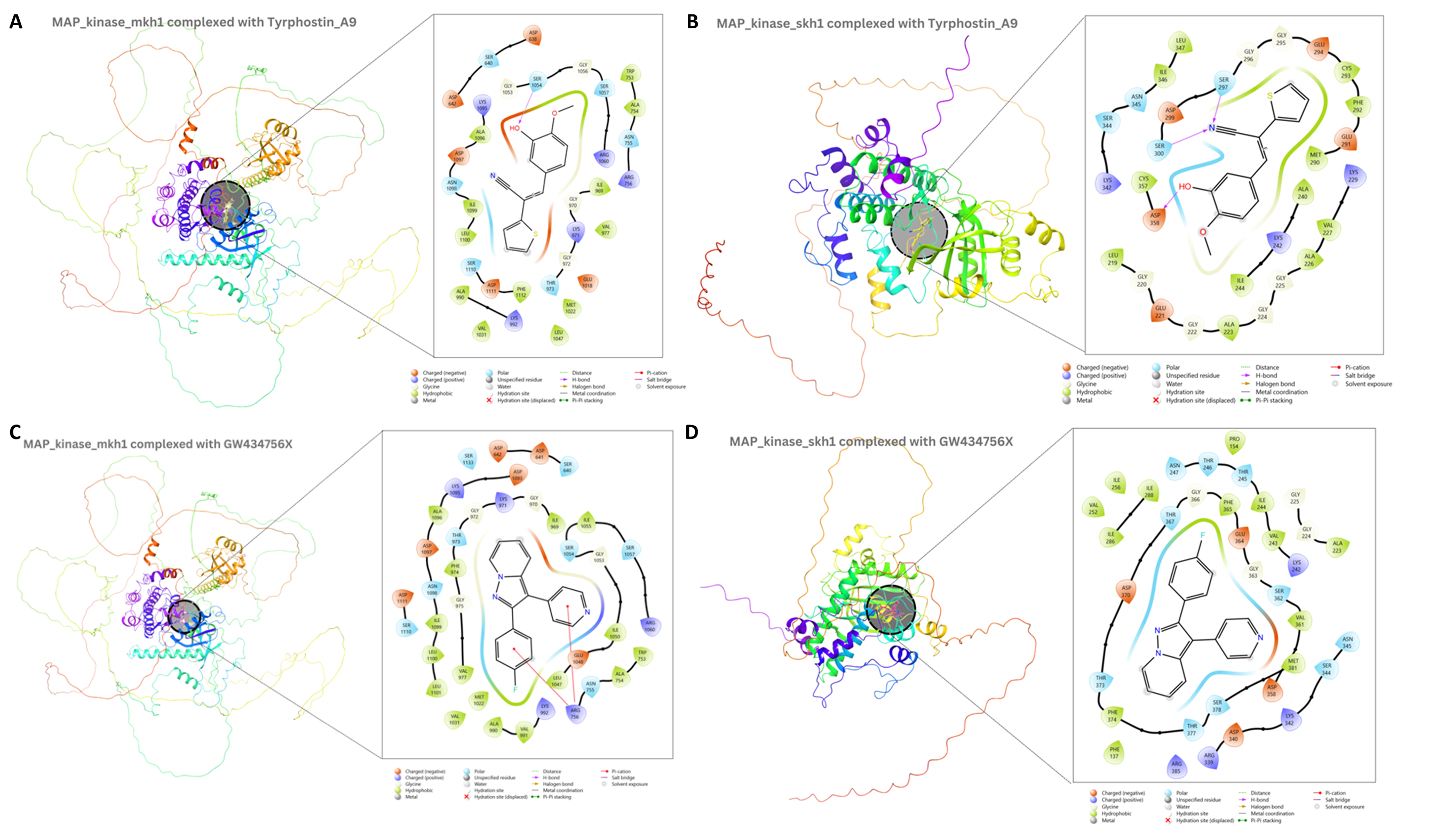

Supplement: S4 Fig — (PNG) [file pntd.0013482.s014.png]
